# Supplementary material for: Prediction of Pharmacokinetics for CYP3A4-Metabolized Drugs in Pediatrics and Geriatrics Using Dynamic Age-Dependent Physiologically Based Pharmacokinetic Models
Source: Pharmaceutics. 2025 Feb 7;17(2):214. doi: 10.3390/pharmaceutics17020214 (PMC11860008; doi:10.3390/pharmaceutics17020214)
Supplement: Supplementary file 1 [file pharmaceutics-17-00214-s001.zip › supplementary File S1.pdf]

Supplementary Information

Prediction of Pharmacokinetics for CYP3A4-Metabolized Drugs in Pediatrics and Geriatrics Using Dynamic Age-Dependent Physiologically Based Pharmacokinetic Models

Jing Han †, Zexin Zhang †, Xiaodong Liu, Hanyu Yang * and Li Liu *

Center of Drug Metabolism and Pharmacokinetics, School of pharmacy, China Pharmaceutical University, Nanjing 210009, China; 3222010272@stu.cpu.edu.cn (J.H.); 1821010211@stu.cpu.edu.cn (Z.Z.); xdliu@cpu.edu.cn (X.L.)

* Correspondence: shenyhy@cpu.edu.cn (H.Y.); liulee@cpu.edu.cn (L.L.); Tel.: +86-13914732571 (L.L.)

† These authors contributed equally to this work.

1. Development of dynamic age-dependent PBPK model

A dynamic age-dependent PBPK model (Figure S1) was developed to describe the pharmacokinetic profiles of midazolam, fentanyl, alfentanil and sufentanil in plasma of human at all stages of ages following oral or intravenous administrations.

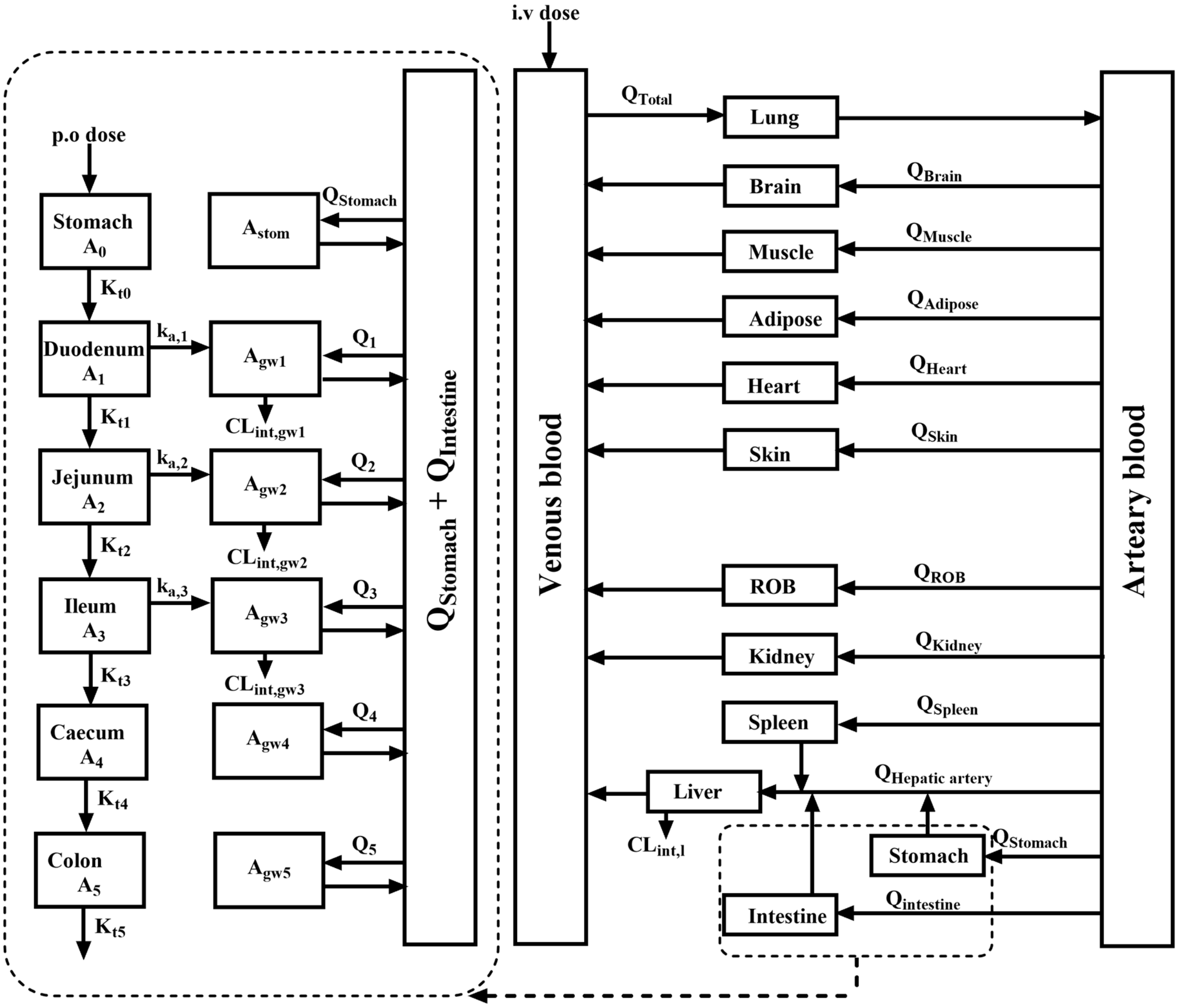

Figure S1. PBPK model for simultaneously illustrating pharmacokinetics of midazolam, fentanyl, alfentanil and sufentanil in adults, pediatrics and geriatrics. Q, tissue blood flow; $CL_{int,l}$, hepatic intrinsic clearance; gwi, intestinal wall; $K_{t,i}$, the transit rate constant; $k_{a,i}$, drug absorption rate constant from intestinal lumen; ROB, rest of body (other tissue).

The dynamic age-dependent PBPK model consists of stomach, intestine, liver, spleen, kidneys, muscle, adipose tissue, skin, heart, brain, lungs, and other tissue, which are connected by the blood circulatory system. The intestine is divided into duodenum, jejunum, ileum, cecum, and colon according to characteristics of physiology and anatomy. Each intestinal segment is further divided into intestinal wall and lumen. Drugs are mainly eliminated via the liverand kidneys. Drug metabolism also occur at wall of duodenum, jejunum and ileum.

In general tissues, the drug amount(A_t) is illustrated by the equation

$$\frac{dA_t}{dt}=Q_t\times(C_{art}-\frac{A_t/V_t}{K_{t:p}/R_b}) \quad (S1)$$

where V_t, Q_t and C_{art} denote organ volume, blood flow rate in tissue and drug concentration in arterial blood, respectively. $K_{t:p}$ and R_b represent the ratios of drug concentration in tissue to plasma and in blood to plasma, respectively.

In stomach

Assuming that neither absorption nor metabolism occurs in the stomach, the amount of drug in stomach(A_0) is mainly controlled by the rate of gastric emptying($K_{t,0}$)

$$\frac{dA_0}{dt}=-K_{t,0}\times A_0 \quad (S2)$$

In intestinal lumen

The intestinal lumen is divided into duodenum, jejunum, ileum, cecum and colon according to physiological characteristics. Absorption and metabolism of drug only occur in the duodenum, jejunum and ileum.

The amount of drug(A_i) in the intestinal lumen,

$$\frac{dA_i}{dt}=K_{t,i-1}\times A_{i-1}-K_{t,i}\times A_i-k_{a,i}\times A_i \quad (S3)$$

where, the subscripts i=1, 2 and 3 represent duodenum, jejunum and ileum, respectively. $K_{t,i}$ denotes the transit rate constant of the ith intestinal lumen; $k_{a,i}$ denotes the rate constant of drug absorption in the ith intestinal lumen.
$k_{a,i}$ may be estimated from human effective permeability in apical to basolateral direction (P_{eff}) across intestinal wall using equation [1,2].

$$k_{a,i}=\frac{2\times P_{eff}}{r_i} \quad (S4)$$

where r_i denotes the radius of the ith intestinal lumen.

In intestinal wall(A_{gwi})

Wall of small intestine also highly expresses CYP3A4, mediating metabolism of CYP3A substrates. Amount of drug in intestinal wall is illustrated by following equation.

$$\frac{dA_{gwi}}{dt}=\left(C_{art}-\frac{A_{gwi}/V_{gwi}}{K_{gut:p}/R_b}\right)\times Q_{gwi}+k_{a,i}\times A_i-f_{u,gut}\times CL_{int,gwi}\times\frac{A_{gwi}/V_{gwi}}{K_{gut:p}/R_b} \quad (S5)$$

where Q_{gwi} and V_{gwi} denote the rate of blood flow and volume of the ith intestinal region. $CL_{int,gwi}$ is CYP3A-mediated intrinsic clearance of drug in intestinal wall. Levels of CYP3A protein in duodenum, jejunum and ileum were reported to be 9.7 nmol, 38.4 nmol and 22.4 nmol, respectively. The $CL_{int,gwi}$ was scaled based on content of CYP3A in intestine [3] and liver [4]. $f_{u,gut}$ was the unbound drug concentration which was defaulted to 1 [5,6] for oral administration and equaled to free fraction of drug($f_{u,b}$) in blood for intravenous administration. Q_{st}

In liver(A_{liv})

$$\frac{dA_{liv}}{dt}=Q_{liv}\times C_{art}-Q_l\times\frac{A_{liv}/V_{liv}}{K_{liv:p}/R_b}+Q_{st}\times\frac{A_{st}/V_{st}}{K_{st:p}/R_b}+Q_{sp}\times\frac{A_{sp}/V_{sp}}{K_{sp:p}/R_b}+\sum_{i=0}^{5}(Q_{gwi}\times\frac{A_{gwi}/V_{gwi}}{K_{gut:p}/R_b})-PBSF\times f_{u,b}\times CL_{int,adult}\times\frac{A_{liv}/V_{liv}}{K_{liv:p}/R_b} \quad (S6)$$

where sp and st denote spleen and stomach, respectively. PBSF is total hepatic microsomal protein amount. $f_{u,b}$ is free fraction of drug in blood, which was estimated using the equation $f_{u,b}=f_{u,p}*(1-Hct)/R_b$, where $f_{u,p}$ is free fraction of drug in plasma. Hct (42%) [7] and R_b is the hematocrit and ratio of drug concentration in blood to plasma, which were assumed the same in different age group. Q_l and Q_{liv} are the blood flow rate of total liver and liver artery, the former equals to the sum of Q_{sp}, Q_{st}, Q_{liv} and Q_{gwi}. $CL_{int,adult}$ is the intrinsic hepatic clearance of the drugs (mL/min/mg) in adult, which can be estimated using hepatic microsomes experiment or recalculated from total hepatic clearance (CL_l) using

Equation S7.

$$CL_l=\frac{Q_l\times(f_{u,b}/R_b)\times CL_{int,adult}\times PBSF}{Q_l+(f_{u,b}/R_b)\times CL_{int,adult}\times PBSF} \tag{S7}$$

In arterial blood(A_{art})

$$\frac{dA_{art}}{dt}=\left(\frac{A_{lun}/V_{lun}}{K_{lun:p}/R_b}-A_{art}/V_{art}\right)\times Q_{total} \tag{S8}$$

In mixed vein (A_{ven})

$$\frac{dA_{ven}}{dt}=\sum Q_t\times\frac{A_t/V_t}{K_{t:p}/R_b}-Q_{total}\times A_{ven}/V_{ven} \tag{S9}$$

where Q_{total} denotes cardiac output. V_{art} and V_{ven} are volume of arterial blood and venous blood, respectively.

In lung(A_{lun})

$$\frac{dA_{lun}}{dt}=\left(A_{ven}/V_{ven}-\frac{A_{lun}/V_{lun}}{K_{lun:p}/R_b}\right)\times Q_{total} \tag{S10}$$

2. List of Tables

Table S1 Pharmacokinetic parameters of midazolam, fentanyl, alfentanil and sufentanil used in the simulation

	Midazolam	Fentanyl	Alfentanil	Sufentanil
$K_{t:p}$				
Adipose	9.23	170.39	8.22	114.55
Liver	4.10	4.55	0.28	2.95
Muscle	1.33	2.76	0.19	1.75
Lung	4.61	5.45	0.32	3.57
Kidney	4.72	4.14	0.26	2.69
Brain	3.38	8.22	0.46	5.41
Heart	4.31	3.75	0.24	2.43
Spleen	3.38	2.54	0.18	1.60
Skin	1.44	12.46	0.65	8.31
Intestine	4.92	8.63	0.47	5.70
ROB	0.001[a]	0.001[a]	0.001[a]	0.001[a]
Stomach	7.79	8.63	0.47	5.70
pKa	/	8.43 [8]	6.5 [9,10]	8 [11]
logP	/	4.05 [12]	2.1 [13]	3.95 [11]
$f_{u,p}$	0.044 [14]	0.2084 [15]	0.086 [13]	0.075 [11]
R_b	0.6 [3]	1 [12]	0.63 [16]	0.74 [11]
P_{eff}(cm/min)	0.0264 [3]	/	/	/
CL_{int}(mL/min/mg.protein)	0.389 [17]	0.0783 [18]	0.096 [19]	0.934 [20]

pK_a, acid dissociation constant; log P, the log of the partition coefficient of a solute between octanol and water; $f_{u,p}$, fraction unbound in plasma; R_b, blood to plasma ratio; P_{eff}, effective permeability coefficient; $CL_{int,l}$, CYP3A4 enzyme-mediated intrinsic hepatic clearance; ROB, rest of body; The $K_{t:p}$ values of fentanyl, alfentanil and sufentanil were calculated using the method reported [21]; the $K_{t:p}$ values of midazolam were cited from $K_{t:p}$ values of rats [22], assuming that the ratios of tissue concentrations and free plasma concentrations in human were equaled to those in rats. The Cl_{int} of fentanyl and sufentanil was calculated from in vivo hepatic clearance using Equation S7.

Table S2. Physiological parameters used in adults

Tissue	Volume (mL) [23]	Blood flow (mL/min) [23]	Transit rate constant (min^{-1}) [23]	Intestinal radius (cm) [23]	PBSF (mg microsome protein)
Lung	1170	5600	/	/	/
Heart	310	240	/	/	/
Brain	1450	700	/	/	/
Muscle	35000	750	/	/	/
Adipose	10000	260	/	/	/
Skin	7800	300	/	/	/
Kidneys	280	1240	/	/	/
Spleen	190	80	/	/	/
Liver	1690	1518.33	/	/	/
Vein	3470	/	/	/	/
Artery	1730	/	/	/	/
ROB	5100	592	/	/	/
Stomach	160	38.33	0.08	/	/
Duodenum	70	118	0..07	/	/
Jejunum	209	413	0.03	/	/
Ileum	139	244	0.04	/	/
Cecum	116	44	0.003	/	/
Colon	1116	281	0.001	/	/
r_1	/	/	/	2	/
r_2	/	/	/	1.63	/
r_3	/	/	/	1.45	/
PBSF	/	/	/	/	64220

PBSF can be calculated by multiplying the liver weight (g) and microsomal protein yield (mg protein/g liver weight), which is 38 mg [24,25].

Table S3. Equations of tissue/organ volume with age (A, years) for pediatrics

Tissue weight (g)	Equations
Lungs [26]	$-0.0346\times A^4+1.5069A^3-20.31A^2+123.99A+59.231$
Heart [26]	$-0.0132\times A^4+0.5051\times A^3–5.7113\times A^2+32.213\times A+20.364$
Brain [26]	$10^4\times(A+0.213)/(6.030+6.895\times A)$
Muscle [27]	$0.535\times A^3+56.937\times A^2-124.25\times A+1051.3$
Adipose [26]	$0.0165\times A^5-1.9784\times A^4+51.963\times A^3-459.38\times A^2+1566.8\times A+1004.2$
Skin [27]	$-0.0992\times A^4+4.2762\times A^3-62.165\times A^2+432.78\times A+203.2$
Kidney [26]	$9.737\times 10^{-4}\times A^5-0.0561\times A^4+1.1729\times A^3-10.34\times A^2+44.604\times A+28.291$
Spleen [26]	$-0.0091\times A^4+0.3457\times A^3-4.0754\times A^2+22.269\times A+11.05$
Stomach [26]	$0.0008\times A^5-0.0356\times A^4+0.5823\times A^3-4.0437\times A^2+17.888\times A+7.54$
Liver [26]	$0.0072\times A^5-0.3975\times A^4+7.9052\times A^3-65.624\times A^2+262.02\times A+157.52$
Intestinal tract [26]	$-0.047817\times A^4+1.925\times A^3-22.382\times A^2+107.09\times A+51.125$
Blood Volume [26]	$-0.0623\times A^5+2.4425\times A^4-31.37\times A^3+149.98\times A^2+31.305\times A^2+393.7$

Assuming an average specific gravity of 1 g/mL.

Table S4. Equations of blood flow in tissues/organs with age (A,years) for pediatrics

Blood flow rate	Equations
cardiac output(L/h) [26]	$0.012\times A^3-1.2144\times A^2+40.324\times A+44.414$
Heart (mL/min) [28]	$0.79*W_{heart}^a$
Brain (L/h) [26]	$-0.0024\times A^4+0.1305\times A^3-2.4822\times A^2+18.025\times A+15.197$
Muscle (mL/min) [29]	$(4.73-0.189\times A)/(1+0.0672\times A-0.00515\times A^2)$
Adipose (mL/min) [28]	$0.024*W_{adipose}^a$
Skin (mL/min) [29]	$(9.65+25.7\times A)/(1+1.04\times A+0.0585\times A^2)$
Kidney (mL/min) [29]	$(229+700\times A)/(1+1.49\times A+0.0108\times A^2)$
Spleen (mL/min) [29]	$(223+102\times A)/(1+1.03\times A+0.004\times A^2)$
Stomach (mL/min) [29]	$(128+63.9\times A)/(1+1.11\times A+0.00568\times A^2)$
Liver(mL/min) [29]	$(25.6+2.33\times A)/(1-0.0227\times A+0.00508\times A^2)$
Intestinal tract(mL/min) [29]	$(216+106\times A)/(1+1.09\times A+0.00562\times A^2)$

a Wheart and Wadipose are organ weight of heart and adipose, which are listed in Table S3

Table S5. Coefficients(a, b, c and d) of weight and height related to age (years) for pediatics [29]

	**0-2 years**				**2-20 years**			
	**a**	**b**	**c**	**d**	**a**	**b**	**c**	**d**
weight	3.41	20.1	1.46	−0.107	9.86	0.370	−0.0789	0.00205
height	50.5	117	1.28	−0.0696	74.8	3.35	−0.0354	0.00125

Table S6 Equations of tissue/organ volume with age (A, years) for geriatrics [30]

Tissues/volume	Equations
Height (H, cm)	$-0.0039\times A^2+0.238\times A-12.5\times Sex+176$
Body weight (BW, kg)	$-0.0039\times A^2+1.12\times H+0.611\times A-0.424\times Sex-137$
Lung (kg)	$e^{(0.028\times H+0.0077\times A-5.6)}$
Adipose tissue (kg)	$0.68\times BW-0.56\times H+6.1\times Sex+65$
Brain (kg)	$e^{(-0.0075\times A+0.0078\times H-0.97)}$
Heart (kg)	$0.34\times BSA+0.0018\times A-0.36$
Kidney (kg)	$-0.00038\times A-0.056\times Sex+0.33$
Muscle (kg)	$17.9\times BSA-0.0667\times A-5.68\times Sex-1.22$
Skin (kg)	$e^{(-0.0058\times A-0.37\times Sex+1.13)}$
Stomach (g)	160^a
Intestine (kg)	$3\times 10^{-6}\times H^{2.49}$
Spleen (kg)	$e^{(1.13\times BSA-3.93)}$
Liver (kg)	$e^{(0.87\times BSA-0.0014\times A-1)}$
Blood (kg)	$e^{(0.067\times BSA-0.0025\times A-0.38\times Sex+1.7)}$

*Male sex=0. Female sex=1. BSA were calculated from Equation (3) in manuscript (BW >15kg)

a Assuming the organ weight of stomach was the same in geriatrics and adults.

Table S7. Equations of blood flow in tissues/organs with age (A, years) for geriatrics [30]

**Blood flow**	**Equations**
Cardiac output (CO,L/h)	159×BSA-1.56×A+114
Adipose tissue (CO%)	(0.044+0.027×Sex)×A+2.4×Sex+3.9
Brain (CO%)	$e^{(-0.48\times BSA+0.044\times Sex+3.5)}$
Heart (CO%)	-0.72×H-10×Sex+134
Kidney (CO%)	-8.7×BSA+0.29×H-0.081×A-13
Muscle (CO%)	-6.4×Sex+17.5
Skin (CO%)	5
Stomach (mL/min)	38.33[a]
Intestine (CO%)	2×Sex+14
Spleen (CO%)	3
Liver (CO%)	-0.108×A+1.04×Sex+27.9

*Male sex=0. Female sex=1. BSA were calculated from Equation (5) in manuscript (BW >15kg)

a Assuming the blood flow of stomach was the same in geriatrics and adults.

Table S8 Subject's characteristics for midazolam clinical data

No.	Research	Drug (base mg)	Dose (base mg)	Age(year)	Weight(kg)	Subjects' information	Subjects number	Ref.
1	Lee et al. 1996	Midazolam,infusion	0.1mg/kg(2min)	GA 24-30w	0.571-1.418	NA	15	[31]
2	Mulla et al. 2003	Midazolam,infusion	259ug/kg/h(148.8h)	GA 39.5±1.9w + PNA 0.5-18d	3.4±0.6	NA	20	[32]
3	Mulla et al. 2003	Midazolam,infusion	259ug/kg/h(148.8h)	GA 39.5±1.9w + PNA 0.5-18d	3.4±0.6	minor otolaryngological surgery	1	[32]
4	Malinovsky et al. 1993	Midazolam,infusion	0.2mg/kg	2-7	19.4±5.1	Healthy volunteer- CC genotype	15	[33]
5	Walbergh et al. 1991	Midazolam,infusion	0.1mg/kg	3.0±1.4	12.8±3.3	Healthy volunteer- CT genotype	9	[34]
6	Rey et al. 1991	Midazolam,infusion	0.2mg/kg	2.5±0.8	15.2±1.2	Healthy volunteer- TT genotype	6	[35]
7	Salonen et al. 1987	Midazolam,infusion	0.075mg/kg	7.33±6.66	32.8±26.3	Healthy volunteer- CC genotype	3	[36]
8	Salonen et al. 1987	Midazolam,infusion	0.15mg/kg	6.72±5.94	28±22.7	Healthy volunteer- CT genotype	6	[36]
9	Salonen et al. 1987	Midazolam,infusion	0.3mg/kg	6.07±4.59	21.6±13.7	Healthy volunteer- TT genotype	6	[36]
10	Salonen et al. 1987	Midazolam,infusion	0.45mg/kg	6.11±1.67	22.3±3.19	Healthy volunteer	6	[36]
11	Tolia et al. 1991	Midazolam,infusion	0.08mg/kg(5min)	8-17	NA	Healthy volunteer	20	[37]
12	Payne et al. 1989	Midazolam,infusion	0.15mg/kg	5.52±1.87	17.3±4.43	Healthy volunteer	8	[38]
13	Payne et al. 1989	Midazolam,oral	0.15mg/kg	5.81±1.63	17.5±5.17	Healthy volunteer	8	[38]
14	Payne et al. 1989	Midazolam,oral	0.45 mg/Kg	5.48±1.63	17.2±3.91	Healthy volunteer	8	[38]
15	Payne et al. 1989	Midazolam,oral	1mg/kg	5.97±1.73	17.8±3.79	Healthy volunteer	8	[38]
16	Jones et al. 1994	Midazolam,infusion	0.5mg/kg(0.5min)	4-11	15-27	orthopaedic surgery	10	[39]
17	Reed et al. 2001	Midazolam,infusion	0.15mg/kg(2-3min)	4.6±1.8	17.7±3.7	Healthy volunteer	12	[40]
18	Reed et al. 2001	Midazolam,oral	0.25mg/kg	7.3±2.3	28.6±10.9	Healthy volunteer	23	[40]

w means week, m means month and d means day.

Continued Table S8

No.	Research	Drug (base mg)	Dose (base mg)	Age(year)	Weight(kg)	Subjects' information	Subjects number	Ref.
19	Reed et al. 2001	Midazolam,oral	0.5mg/kg	7.3±2.3	28.6±10.9	NA	21	[40]
20	Reed et al. 2001	Midazolam,oral	1mg/kg	7.3±2.3	28.6±10.9	NA	23	[40]
21	Hiller et al. 1990	Midazolam,oral	0.5mg/kg	7.7±1.1	28±2.4	minor otolaryngological surgery	6	[41]
22	Brosius et al. 2003	Midazolam,oral	0.5mg/kg	5.3±1.9	20.0±6.0	surgical procedures with minimal anticipated blood loss	25	[42]
23	Brosius et al. 2003	Midazolam,oral	0.5mg/kg	5.1±1.9	19.1±5.9	surgical procedures with minimal anticipated blood loss	25	[42]
24	Yang et al. 2011	Midazolam,oral	7.5mg	20-24	60-75	Healthy volunteer- CC genotype	7	[43]
25	Yang et al. 2011	Midazolam,oral	7.5mg	21-28	57-72	Healthy volunteer- CT genotype	8	[43]
26	Yang et al. 2011	Midazolam,oral	7.5mg	20-26	62-68	Healthy volunteer- TT genotype	7	[43]
27	Yang et al. 2011	Midazolam,infusion	5mg	20-24	60-75	Healthy volunteer- CC genotype	7	[43]
28	Yang et al. 2011	Midazolam,infusion	5mg	21-28	57-72	Healthy volunteer- CT genotype	8	[43]
29	Yang et al. 2011	Midazolam,infusion	5mg	20-26	62-68	Healthy volunteer- TT genotype	7	[43]
30	Misaka et al. 2010	Midazolam,infusion	5ug/kg	22.4±6.4	65.5±9.3	Healthy volunteer	5	[44]
31	Misaka et al. 2010	Midazolam,infusion	15ug/kg	22.4±6.4	65.5±9.3	Healthy volunteer	5	[44]
32	Misaka et al. 2010	Midazolam,infusion	30ug/kg	22.4±6.4	65.5±9.3	Healthy volunteer	5	[44]
33	Misaka et al. 2010	Midazolam,oral	15ug/kg	22.4±6.4	65.5±9.3	Healthy volunteer	5	[44]
34	Misaka et al. 2010	Midazolam,oral	50ug/kg	22.4±6.4	65.5±9.3	Healthy volunteer	5	[44]
35	Misaka et al. 2010	Midazolam,oral	100ug/kg	22.4±6.4	65.5±9.3	Healthy volunteer	5	[44]
36	Hase et al. 1997	Midazolam,infusion	0.2mg/kg	17-40	58±11	orthopaedic surgery	15	[45]
37	Yan et al. 2008	Midazolam,infusion	5ug/kg	23±2.3	59.5±9.4	Healthy volunteer	14	[46]
38	Yan et al. 2008	Midazolam,oral	15ug/kg	23±2.3	59.5±9.4	Healthy volunteer	10	[46]

Continued Table S8

No.	Research	Drug (base mg)	Dose (base mg)	Age(year)	Weight(kg)	Subjects' information	Subjects number	Ref.
39	Wermeling et al. 2009	Midazolam,infusion	2.5mg(15min)	20-29	60-92	Healthy volunteer	18	[47]
40	Wermeling et al. 2006	Midazolam,infusion	5mg(15min)	20-29	60-92	Healthy volunteer	12	[48]
41	Ibrahim et al. 2002	Midazolam,infusion	0.07 mg/kg(5min)	31±5	75±13	Healthy volunteer	12	[49]
42	Syed et al. 2012	Midazolam,infusion	1.25mg	25-53	58.1-90.8	Healthy volunteer	24	[50]
43	Syed et al. 2012	Midazolam,oral	5mg	25-53	58.1-90.8	Healthy volunteer	24	[50]
44	Clausen et al. 1988	Midazolam,infusion	0.3mg/kg	23-32	68±6	Healthy volunteer	8	[51]
45	Shao et al.2017	Midazolam maleate,oral	7.5mg(day1)	23.7±2.3	62±5.2	Healthy volunteer-	3	[52]
46	Shao et al.2017	Midazolam maleate,oral	7.5mg(day22)	23.7±2.3	62±5.2	Healthy volunteer-	3	[52]
47	Bornemann et al. 1985	Midazolam,oral	7.5mg	24-52	54.9-92.5	Healthy volunteer	12	[53]
48	Bornemann et al. 1985	Midazolam,oral	15mg	24-52	54.9-92.5	Healthy volunteer	12	[53]
49	Bornemann et al. 1985	Midazolam,oral	30mg	24-52	54.9-92.5	Healthy volunteer	12	[53]
50	Greenblatt et al. 2009	Midazolam,oral	3mg	21-50	52-97	Healthy volunteer	13	[54]
51	olkkola et al. 1993	Midazolam,oral	15mg	18-29	50-73	Healthy volunteer	12	[55]
52	olkkola et al. 1993	Midazolam,infusion	0.05mg/kg(2min)	20-22	56-70	Healthy volunteer	6	[55]
53	olkkola et al. 1996	Midazolam,oral	7.5mg (day1)	19-25	57-95	Healthy volunteer	12	[56]
54	olkkola et al. 1996	Midazolam,oral	7.5mg (day6)	19-25	57-95	Healthy volunteer	12	[56]
55	olkkola et al. 1996	Midazolam,infusion	0.05mg/kg(2min)	19-25	57-95	Healthy volunteer	12	[56]
56	Palkama et al. 1999	Midazolam,oral	7.5mg	21-32	49-88	Healthy volunteer	12	[57]

Continued Table S8

No.	Research	Drug (base mg)	Dose (base mg)	Age(year)	Weight(kg)	Subjects' information	Subjects number	Ref.
57	Palkama et al. 1999	Midazolam,infusion	0.05mg/kg(2min)	21-32	49-88	Healthy volunteer	25	**[57]**
58	Saari et al. 2005	Midazolam,oral	7.5mg	23-29	65-100	Healthy volunteer	20	**[58]**
59	Saari et al. 2005	Midazolam,infusion	0.05mg/kg(2min)	23-29	65-100	Healthy volunteer	6	**[58]**
60	Link et al.2008	Midazolam,oral	7.5mg	21-46	60-78	Healthy volunteer	6	**[59]**
61	Link et al.2008	Midazolam,infusion	2mg	21-46	60-78	Healthy volunteer	5	**[59]**
62	Smith et al. 1981	Midazolam,oral(tablet)	10mg	21-22	66.25-77.9	Healthy volunteer	9	**[60]**
63	Smith et al. 1981	midazolam maleate ,infusion	5mg	21-22	66.25-77.9	Healthy volunteer	1	**[60]**
64	Smith et al. 1981	Midazolam,oral(solution)	10mg	21-22	66.25-77.9	Healthy volunteer	8	**[60]**
65	Wang et al.2000	Midazolam,oral	15mg	22-27	57-90	Healthy volunteer	8	**[61]**
66	Wang et al. 2016	Midazolam,oral	15mg	23-43	61.5±8.6	Healthy volunteer	8	**[62]**
67	Wang et al. 2016	Midazolam,oral	15mg	55-62	73.9±13.4	Healthy volunteer	8	**[62]**
68	castleden et al.1987	Midazolam,oral	15mg	22-37	62.2±7.2	Healthy volunteer	10	**[63]**
69	Greenblatt et al. 1984	Midazolam,oral	10mg	32	NA	Healthy volunteer	12	**[64]**

Continued Table S8

No.	Research	Drug (base mg)	Dose (base mg)	Age(year)	Weight(kg)	Subjects' information	Subjects number	Ref.
70	Greenblatt et al. 1984	Midazolam,infusion	5mg（10min）	32	NA	NA	23	[64]
71	Greenblatt et al. 1984	Midazolam,oral	10mg	32	61	NA	21	[64]
72	Greenblatt et al. 1984	Midazolam,infusion	5mg（10min）	32	61	NA	23	[64]
73	Greenblatt et al. 1984	Midazolam,oral	10mg	36	136	minor otolaryngological surgery,3male,3female	6	[64]
74	Greenblatt et al. 1984	Midazolam,infusion	5mg（10min）	36	136	Endoscopy,9male,4female	6	[64]
75	Platten et al. 1998	Midazolam,infusion	0.05mg/kg	30±8	60-90	Endoscopy	6	[65]
76	Smith et al. 1984	Midazolam,infusion	0.07mg/kg	71.3±6.6	61.46±11.24	Endoscopy	6	[66]
77	Smith et al. 1984	Midazolam,infusion	0.07mg/kg	71.3±6.6	61.46±11.24	Healthy volunteer	NA	[66]
78	Smith et al. 1984	Midazolam,oral	10mg	71.3±6.6	61.46±11.24	Healthy volunteer	NA	[66]
79	castleden et al.1987	Midazolam,oral	15mg	65-80	64.1±15.7	Healthy volunteer,1male	1	[63]
80	Greenblatt et al. 1984	Midazolam,oral	10mg	70	NA	Healthy volunteer,1male	1	[64]
81	Greenblatt et al. 1984	Midazolam,infusion	5mg（10min）	70	NA	Healthy volunteer,1male	1	[64]
82	Platten et al. 1998	Midazolam,infusion	0.03mg/kg	69±5	44-87	Healthy volunteer,1male	1	[65]
83	Quinney et al. 2008	Midazolam,infusion	0.05mg/kg(30min)	66-80	56-112	Healthy volunteer,1female	1	[67]
84	Quinney et al. 2008	Midazolam,oral	3.5mg	66-80	56-112	Healthy volunteer,1female	1	[67]
85	Krupka et al. 2006	Midazolam,oral	5mg	65-71	60-69	Healthy volunteer,1female	1	[68]

Table S9 Subject's characteristics for fentanyl clinical data

No.	Research	Drug (base mg)	Dose (base mg)	Age	Weight	Subjects' information	Subjects' number	Ref.
1	Singleton et al. 1987	fentanyl,infusion	31.2μg/kg(2min)	3-10m	NA	nonabdominal surgery	50	[68,69]
2	Singleton et al. 1987	fentanyl infusion	30.8μg/kg(2min)	1-9yr	NA	nonabdominal surgery	5	[68,69]
3	Dsida et al. 1998	fentanyl infusion	2μg/kg	5.6±2.7	28±2.7	elective tonsillectomy	1	[70]
4	Gauntlett et al. 1988	fentanyl infusion	52.5μg/kg(2min)	GA 38w + PNA 1 d	2.8	Omphalocele repair	1	[71]
5	Gauntlett et al. 1988	fentanyl infusion	56.5μg/kg(2min)	GA 40w + PNA 3 d	2.5	Hydronephrosis repair	1	[71]
6	Saarenmaa et al. 2000	fentanyl infusion	10.5μg/kg over 1h followed by 1.5 μg/kg/h for 58 h	GA 32w + PNA 10h	1.753	Intensive Care Unit	1	[72]
7	Koehntop et al. 1986	fentanyl infusion	25μg/kg	PNA:2d	2	Septic ileus	16	[73]
8	Koehntop et al. 1986	fentanyl infusion	50μg/kg	PNA:2d	3.5	Omphalocele	6	[73]
9	Koehntop et al. 1986	fentanyl infusion	50μg/kg	PNA:1d	3.2	Myelorneningocele	7	[73]
10	Koehntop et al. 1986	fentanyl infusion	25μg/kg	PNA:0.5d	3.5	Myelorneningocele	7	[73]
11	Koehntop et al. 1986	fentanyl infusion	50μg/kg	PNA:0.5d	4	Myelorneningocele	7	[73]
12	Koehntop et al. 1986	fentanyl infusion	25μg/kg	PNA:0. 5d	2.6	Omphalomesenteric duct	7	[73]
13	Rauck et al. 2017	fentanyl infusion	50mg	33.4±8.52	76.92±13.80	healthy volunteers	7	[74]
14	McClain et al. 1980	fentanyl infusion	6.4mg/kg	22-29	75.4±3.7	healthy volunteers	3	[75]
15	McClain et al. 1980	fentanyl infusion	6.4mg/kg	22-29	70.5	healthy volunteers	2	[75]
16	McClain et al. 1980	fentanyl infusion	6.4mg/kg	22-29	82.7	healthy volunteers	5	[75]
17	McClain et al. 1980	fentanyl infusion	6.4mg/kg	22-29	84.5	healthy volunteers	5	[75]
18	McClain et al. 1980	fentanyl infusion	6.4mg/kg	22-29	65	healthy volunteers	4	[75]
19	Ziesenitz et al. 2015	fentanyl infusion	5mg/kg	32.7±8.8	73.4±9.4	healthy volunteers	1	[76]

Continued Table S9

No.	Research	Drug (base mg)	Dose (base mg)	Age	Weight	Subjects' information	Subjects number	Ref.
20	Nozari et al. 2019	fentanyl infusion	200μg	53±17.6	80.1±4.2	craniotomy	6	[77]
21	Singleton et al. 1988	fentanyl infusion	20.7μg/kg(2min)	18-41	NA	elective nonabdominal surgery	7	[78]
22	Bovill et al. 1980	fentanyl infusion	60μg/kg(2min)	55.67±8.22	69±13.95	elective cardiac surgery with cardiopulmonary bypass	3	[79]
23	Bovill et al. 1980	fentanyl infusion	60μg/kg(2min)	58±6	74.5±4.5	elective cardiac surgery with cardiopulmonary bypass	2	[79]
24	Bovill et al. 1980	fentanyl infusion	60μg/kg(2min)	56.6±7.5	71.2±11.5	elective cardiac surgery with cardiopulmonary bypass	5	[79]
25	Bentley et al. 1982	fentanyl infusion	10μg/kg	36±4	64±3	NA	5	[80]
26	Bentley et al. 1982	fentanyl infusion	10μg/kg	33	NA	NA	1	[80]
27	Pesonen et al. 2009	fentanyl infusion	7.5μg/kg followed by 0.1μg/kg/min(222min)	52±6	81±14	coronary by-pass grafting with a cardiopulmonary bypass	20	[81]
28	Egan et al. 2000	fentanyl infusion	15μg/kg(22.3min)	27.8±3.8	74.4±11.1	healthy volunteers	12	[82]
29	Scott et al. 1985	fentanyl infusion	150μg/min (5min)	59±6	88±12	elective surgery involving minimal blood loss	6	[83]
30	Christrup et al. 2008	fentanyl infusion	100μg	23.86±1.81	68.29±10.96	surgical removal of both mandibular third molars	7	[84]
31	Duthie et al. 1986	fentanyl infusion	100μg/h from 0 to 24h,a bolus dose of 100 ug was given i.v. at 2 h	58±11	69±11	Prolonged surgery	10	[85]
32	Duthie et al. 1986	fentanyl infusion	100μg/h from 0 to 24h,a bolus dose of 100 ug was given i.v. at 2 h	49±14	65±14	Upper abdominal surgery	13	[85]

Continued Table S9

No.	Research	Drug (base mg)	Dose (base mg)	Age	Weight	Subjects' information	Subjects' number	Ref.
33	Duthie et al. 1986	fentanyl infusion	100μg/h from 0 to 26h,a bolus dose of 100 ug was given i.v. at 2 h	55±12	69±9	Cardiac surgery	12	[85]
34	Holley et al. 1988	fentanyl infusion	loading dose 100μg followed by 25μg/h for 24h	54±12	76±12	Lumbar fusion (2), knee replacement (2), shoulder capsule repair, laparotomy (4), thoractomy	10	[86]
35	Holley et al. 1988	fentanyl citrate infusion	loading dose 200μg followed by 50μg/h for 24h	44±15	81±16	Lumbar laminectomy (2), lumbar fusion, knee replacement (2), knee arthrotomy (2), open tibial fixation, laparotomy, thoracotomy	10	[86]
36	Holley et al. 1988	fentanyl infusion	loading dose 400μg followed by 100μg/h for 24h	56±12	80±17	Lumbar fusion (2), posterior cervical fusion, knee replacement, laparotomy (5), thoracotomy	10	[86]
37	Holley et al. 1988	fentanyl infusion	loading dose 500μg followed by 125μg/h for 24h	54±12	77±5	Lumbar fusion (2), shoulder capsule repair, open tibial fixation, laparotomy (5)	9	[86]
38	Lim et al. 2012	fentanyl infusion	100μg(5min)	19-32	51.4-100.7	healthy volunteers	22	[87]
39	MacLeod et al. 2012	fentanyl infusion	25μg(5s)	25.3±7.2	73.2±13	healthy volunteers	10	[88]

Continued Table S9

No.	Research	Drug (base mg)	Dose (base mg)	Age	Weight	Subjects' information	Subjects' number	Ref.
40	Stoeckel et al. 1982	fentanyl infusion	0.5mg	22.3±3.2	66±9.6	healthy volunteers	3	[89]
41	Stoeckel et al. 1982	fentanyl infusion	0.5mg	22	60	healthy volunteers	1	[89]
42	Stoeckel et al. 1982	fentanyl infusion	0.5mg	34	50	healthy volunteers	1	[89]
43	Stoeckel et al. 1982	fentanyl infusion	0.5mg	35	63	healthy volunteers	1	[89]
44	Stoeckel et al. 1982	fentanyl infusion	0.5mg	26	58	healthy volunteers	1	[89]
45	streisand et al.1991	fentanyl infusion	15μg/kg（7.65min）	23-31	68-85	healthy volunteers	10	[90]
46	Varvel et al. 1989	fentanyl infusion	150μg/min for 5 min(seven patients) or 6.5min(one patient)	33-57	52-100	NA	8	[91]
47	Hynynen et al. 1986	fentanyl infusion	48μg/kg(10min) followed by 0.3μg/kg/min for 205min, followed by 0.15μg/kg/min for 45min	49±12	86±10	coronary artery bypass grafting	9	[92]
48	Ibrahim et al. 2003	fentanyl infusion	5μg/kg(15min)	30±5	74±15	healthy volunteers	12	[93]
49	Singleton et al. 1988	fentanyl infusion	15.5μg/kg(2min)	71-82	NA	elective nonabdominal surgery	7	[78]
50	Bentley et al. 1982	fentanyl infusion	10μg/kg	67±2	68±7	NA	4	[80]
51	Bentley et al. 1982	fentanyl infusion	10μg/kg	65	NA	NA	1	[80]
52	Pesonen et al. 2009	fentanyl infusion	7.5μg/kg,0.1μg/kg/min(203min)	80±14	72±16	coronary by-pass grafting with a cardiopulmonary bypass	30	[81]
53	Duthie et al. 1986	fentanyl infusion	100μg/h from 0 to 24h, a bolus dose of 100 ug was given i.v. at 2 h	61±8	69±12	orthopaedic surgery	10	[85]
54	Hudson et al. 1986	fentanyl infusion	100μg/kg(2min)	67.2±8.7	78.5±13.7	elective abdominal aortic surgery with infrarenal aortic crossclamping	10	[94]

Table S10 Subject's characteristics for alfentanil clinical data

No.	Research	Drug (base mg)	Dose (base mg)	Age	Weight	Subjects' information	Subjects' number	Ref.
1	Roure et al. 1987	alfentanil infusion	20μg/kg	4.97±0.85	NA	elective orthopaedic surgery	18	[95]
2	Pokela et al. 1992	alfentanil infusion	11.8μg/kg(1min)	30-40w	1.49-3.99	critically ill mechanically ventilated	20	[96]
3	Goresky et al. 1987	alfentanil infusion	50μg/kg(0.5min)	3.4±4.1	15.24±10.49	surgery	1	[97]
4	Davis et al. 1989	alfentanil infusion	25μg/kg(0.5min)	GA 29.5±3.3w + PNA:1-3d	0.96-2.4	stressful intensive-care procedures	6	[98]
5	Davis et al. 1989	alfentanil infusion	25μg/kg(0.5min)	5±2.8	NA	stressful intensive-care procedures	9	[98]
6	Helmers et al. 1984	alfentanil infusion	50μg/kg (2min)	27-44	70±16	intra-abdominal surgery	9	[99]
7	Camu et al. 1982	alfentanil infusion	120μg/kg(0.5min)	43±9	57.4±9.7	healthy volunteers scheduled for routine general surgery	5	[100]
8	Kharasch et al. 2001	alfentanil infusion	15μg/kg(0.5min)	19-26	77±6	healthy volunteers	6	[101]
9	Hynynen et al. 1986	alfentanil infusion	48μg/kg(2min) followed by 6μg/kg/min for 215min followed by 3μg/kg/min for 45min	51±6	78±11	coronary artery bypass grafting	10	[92]
10	Ibrahim et al. 2003	alfentanil infusion	15μg/kg(15min)	30±5	74±15	healthy volunteers	12	[93]
11	Fragen et al. 1983	alfentanil infusion	80μg/kg over 30s followed by 3μg/kg/min for 60min	38.8±6.79	72.2±8.16	healthy volunteers	5	[102]
12	Bovill et al. 1982	alfentanil infusion	50μg/kg	42±5.8	73±2	surgical procedures	6	[103]
13	Bovill et al. 1982	alfentanil infusion	125μg/kg	45±9.8	69±5.3	surgical procedures	5	[103]
14	Scott et al. 1985	alfentanil infusion	125μg/kg	49±15	85±6	elective surgery involving minimal blood loss	6	[83]
15	Helmers et al. 1984	alfentanil infusion	50μg/kg (2min)	68-91	66±11	intra-abdominal surgery	15	[99]

Table S11. Subject's characteristics sufentanil clinical data

No.	Research	Drug (base mg)	Dose (base mg)	Age	Weight	Subjects' information	Subjects' number	Ref.
1	Guay et al. 1992	sufentanil infusion	1.74μg/kg	5.13±2.29	18.84±4.61	elective anaesthesia and surgery	7	[104]
2	Guay et al. 1992	sufentanil infusion	2.53μg/kg	5.42±1.06	18.79±5.15	elective anaesthesia and surgery	8	[104]
3	Guay et al. 1992	sufentanil infusion	3.18μg/kg	4.8±1.12	20.1±5.75	elective anaesthesia and surgery	5	[104]
4	Bovill et al. 1984	sufentanil infusion	5μg/kg	45.5±13.73	71.1±18.95	surgery	10	[105]
5	Taverne et al. 1992	sufentanil infusion	150μg	58±13.9	66±8.8	elective major abdominal surgery	10	[106]
6	Helmers et al. 1989	sufentanil infusion	15μg/kg(0.5min)	24-46	65-76	surgery	7	[107]
7	Matteo et al. 1990	sufentanil infusion	2μg/kg	41±15	67±12	elective neurosurgery	7	[108]
8	Zhao et al. 2009	sufentanil infusion	7.2μg/kg	55.8±16.7	53.8±8.8	surgery	12	[109]
9	Matteo et al. 1990	sufentanil infusion	2μg/kg	77±5	58±12	elective neurosurgery	7	[108]
10	Borenstein et al. 1997	sufentanil infusion	30μg/kg/min for 0.5min	60.6±11.6	80.3±11.8	aortocoronary bypass surgery	12	[110]
11	Borenstein et al. 1997	sufentanil infusion	5μg/kg/min for 3min	61.5±10.2	87.3±16.5	aortocoronary bypass surgery	11	[110]
12	Borenstein et al. 1997	sufentanil infusion	2μg/kg/min for 7min	61.3±7.8	79.6±13.7	aortocoronary bypass surgery	9	[110]

Table S12 . Observed and predicted values of AUC_{0-t}, C_{max} of midazolam

No.	age	AUC_{0-t} (ng/mL*min)			C_{max}(ng/mL)			Ref.
		Obs	Pre	Ratio	Obs	Pre	Ratio	
1	GA 24-30w	40708.5 [a]	17519.7 (20.7) [c]	0.4	/	/	/	[31]
2	GA 39.5±1.9w + PNA 0.5-18d	9122259.0 [a]	6795151.7 (25.5) [c]	0.7	/	/	/	[32]
3	2-7	25326.9 [a]	22116.2 (25.3) [c]	0.9	/	/	/	[33]
4	3.0±1.4	4544.8 [a]	5341.5 (18.8) [c]	1.2	/	/	/	[34]
5	2.5±0.8	16980.0±6360.0 (37.5) [b]	11162.6 (15.2) [c]	0.7	/	/	/	[35]
6	7.33±6.66	8280.0±3024.0 (36.5) [b]	10634.8 (23.1) [c]	1.3	/	/	/	[36]
7	6.72±5.94	13920.0±2400.0 (17.2) [b]	14749.0 (25.6) [c]	1.1	/	/	/	[36]
8	6.07±4.59	29160.0±7068.0 (24.2) [b]	27598.5 (25.5) [c]	0.9	/	/	/	[36]
9	6.11±1.67	34560.0±5352.0 (15.5) [b]	41615.5 (25.5) [c]	1.2	/	/	/	[36]
10	8-17	5092.1 [a]	6128.0 (18.8) [c]	1.2	/	/	/	[37]
11	5.52±1.87	16560.0±3804.0 (23.0) [b]	12503.0 (24.8) [c]	0.8	/	/	/	[38]
12	5.81±1.63	4500.0±1428.0 (31.7) [b]	3001.8 (38.4) [c]	0.7	20.9±25.3 (121.1) [b]	27.1 (25.3) [c]	1.3	[38]
13	5.48±1.63	7800.0±2940.0 (37.7) [b]	9044.8 (38.3) [c]	1.2	59.5±23.5 (39.5) [b]	82.0 (25.2) [c]	1.4	[38]
14	5.97±1.73	17160.0±3390.0 (19.8) [b]	20054.4 (38.5) [c]	1.2	82.0±31.6 (38.5) [b]	180.6 (25.4) [c]	2.2	[38]
15	4-11	18344.0 [a]	30146.6 (31.2) [c]	1.6	/	/	/	[39]
16	4.6±1.8	16875.8±6925.3 (41.0) [b]	12432.7 (27.2) [c]	0.7	/	/	/	[40]
17	7.3±2.3	4980.0±1860.0 (37.3) [b]	6175.7 (39.9) [c]	1.2	35.6±19.7 (55.3) [b]	53.9 (26.2) [c]	1.5	[40]
18	7.3±2.3	10860.0±4800.0 (44.2) [b]	12213.6 (39.9) [c]	1.1	70.3±43.2 (61.5) [b]	106.6 (26.2) [c]	1.5	[40]
19	7.3±2.3	23580.0±13200.0 (56.0) [b]	24427.3 (39.9) [c]	1.0	136.6±64.7 (47.4) [b]	213.3 (26.2) [c]	1.6	[40]
20	7.7±1.1	12840.0 (2700.0,23040.0) [d]	12207.9 (42.3) [c]	1.0	73.0 (31.0,114.0) [d]	96.6 (26.7) [c]	1.3	[41]
21	20-24	5678.4±838.2 (14.8) [b]	4606.6 (41.8) [c]	0.8	60.74 [a]	33.4 (27.1) [c]	0.6	[43]

[a]Mean; [b]Mean±SD(CV%); [c]50th percentile of simulated AUC_{0-t}/C_{max}(CV%); [d]Mean(95% confidence interval); [e]Mean(CV%)

Continued Table S12

No.	age	AUC_{0-t} (ng/mL*min)			C_{max}(ng/mL)			Ref.
		Obs	Pre	Ratio	Obs	Pre	Ratio	
22	21-28	5313.6±1227.0 (23.1) [b]	4606.6 (41.8) [c]	0.9	57.6±14.6 (25.3) [b]	33.4 (27.1) [c]	0.6	[43]
23	20-26	5742.6±2065.2 (36.0) [b]	4606.6 (41.8) [c]	0.8	53.4±22.1 (41.5) [b]	33.4 (27.1) [c]	0.6	[43]
24	20-24	11709.6±3000.6 (25.6) [b]	12024.6 (26.4) [c]	1.0	/	/	/	[43]
25	21-28	10999.8±1188.6 (10.8) [b]	12024.6 (26.4) [c]	1.1	/	/	/	[43]
26	20-26	11683.2±1383.0 (11.8) [b]	12024.6 (26.4) [c]	1.0	/	/	/	[43]
27	22.4±6.4	504.0±96.0 (19.0) [b]	603.5 (41.8) [c]	1.2	3.9±0.6 (15.4) [b]	4.4 (27.1) [c]	1.1	[44]
28	22.4±6.4	1812.0±546.0 (30.1) [b]	2011.5 (41.8) [c]	1.1	13.3±2.2 (16.5) [b]	14.6 (27.1) [c]	1.1	[44]
29	22.4±6.4	4350.0±1110.0 (25.5) [b]	4023.1 (41.8) [c]	0.9	26.4±2.3 (8.7) [b]	29.2 (27.1) [c]	1.1	[44]
30	22.4±6.4	3684.0±450.0 (12.2) [b]	5833.3 (25.8) [c]	1.6	/	/	/	[44]
31	22.4±6.4	2070.0±90.0 (4.3) [b]	2957.6 (25.7) [c]	1.4	/	/	/	[44]
32	22.4±6.4	756.0±144.0 (19.0) [b]	985.9 (25.7) [c]	1.3	/	/	/	[44]
33	17-40	25110.0±5814.0 (23.2) [b]	34919.2 (25.7) [c]	1.4	/	/		[45]
34	23±2.3	472.2±234.6 (49.7) [b]	549.3 (42.0) [c]	1.2	3.3±1.1 (32.3) [b]	4.0 (27.1) [c]	1.2	[46]
35	23±2.3	720.0±264.0 (36.7) [b]	905.5 (25.7) [c]	1.3	/	/	/	[46]
36	20-29	6552.0±726.0 (11.1) [b]	5436.9 (27.1) [c]	0.8	/	/	/	[47]
37	20-29	11160.0±1860.0 (16.7) [b]	10684.5 (27.4) [c]	1.0	/	/	/	[48]
38	31±5	11280.0±3660.0 (32.4) [b]	11425.6 (27.1) [c]	1.0	/	/	/	[49]
39	25-53	2580.0 (17.0) [e]	3762.8 (25.7) [c]	1.5	/	/	/	[50]
40	25-53	4320.0 (49.0) [e]	3071.1 (41.8) [c]	0.7	26 (43.0) [e]	22.3 (27.1) [c]	0.9	[50]
41	23-32	48971.0±7521.0 (15.4) [b]	43220.0 (27.8) [c]	0.9	/	/	/	[51]
42	23.7±2.3	7140.0±3600.0 (50.4) [b]	4606.6 (41.8) [c]	0.6	53.8±22.0 (40.9) [b]	33.4(27.1) [c]	0.6	[52]

[a]Mean; [b]Mean±SD(CV%); [c]50th percentile of simulated AUC_{0-t}/C_{max}(CV%); [d]Mean(95% confidence interval); [e]Mean(CV%)

Continued Table S12

No.	age	AUC_{0-t} (ng/mL*min)			C_{max}(ng/mL)			Ref.
		Obs	Pre	Ratio	Obs	Pre	Ratio	
43	23.7±2.3	6240.0±3240.0 (51.9) [b]	4606.6 (41.8) [c]	0.7	42.7±22.7 (53.2) [b]	33.4 (27.1) [c]	0.8	[52]
44	24-52	5520.0±2820.0 (51.1) [b]	4606.6 (41.8) [c]	0.8	34.0±13.0 (38.2) [b]	33.4 (27.1) [c]	1.0	[53]
45	24-52	11280.0±4500.0 (39.9) [b]	9213.2 (41.8) [c]	0.8	67.0±19.0 (28.4) [b]	66.9 (27.1) [c]	1.0	[53]
46	24-52	30180.0±12660.0 (41.9) [b]	18426.3 (41.8) [c]	0.6	212.0±90.0 (42.5) [b]	133.7 (27.1) [c]	0.6	[53]
47	21-50	1479.0±115.8 (7.8) [b]	1844.8 (41.9) [c]	1.2	9.0±1.0 (11.1) [b]	12.7 (27.0) [c]	1.4	[54]
48	18-29	12000.0±1000.0 (8.3) [b]	9213.2 (41.8) [c]	0.8	70.0±9.0 (12.9) [b]	66.9 (27.1) [c]	1.0	[55]
49	20-22	8148.1 [a]	5931.4 (28.7) [c]	0.7	/	/	/	[55]
50	19-25	4680.0±1440.0 (30.8) [b]	4606.6 (41.8) [c]	1.0	28.0±9.0 (32.1) [b]	33.4 (27.1) [c]	1.2	[56]
51	19-25	5220.0±2340.0 (44.8) [b]	4606.6 (41.8) [c]	0.9	35.0±14.0 (40.0) [b]	33.4 (27.1) [c]	1.0	[56]
52	19-25	6454.3 [a]	7155.4 (28.7) [c]	1.1	/	/	/	[56]
53	21-32	7140.0±2580.0 (36.1) [b]	6496.3 (28.7) [c]	0.9	/	/	/	[57]
54	21-32	6480.0±3558.0 (54.9) [b]	4606.6 (41.8) [c]	0.7	26.9±8.6 (32.0) [b]	33.4 (27.1) [c]	1.2	[57]
55	23-29	5460.0±1800.0 (33.0) [b]	4606.6 (41.8) [c]	0.8	24.1±7.2 (29.9) [b]	33.4 (27.1) [c]	1.4	[58]
56	23-29	9060.0±2400.0 (26.5) [b]	7767.3 (28.7) [c]	0.9	/	/	/	[58]
57	21-46	7548.0 (5022.0,16122.0) [d]	6029.8 (25.7) [c]	0.8	/	/	/	[59]
58	21-46	6174.0 (3840.0,9822.0) [d]	4606.6 (41.8) [c]	0.7	63.1 (25.9,80.2) [d]	33.4 (27.1) [c]	0.5	[59]
59	21-22	11418.0±2952.0 (25.9) [b]	11620.2 (26.6) [c]	1.0	/	/	/	[60]
60	21-22	8820.0±4734.0 (53.7) [b]	6142.1 (41.8) [c]	0.7	49.7 [a]	44.6 (27.1) [c]	0.9	[60]
61	21-22	8136.0±3300.0 (37.4) [b]	6142.1 (41.8) [c]	0.8	64.1 [a]	44.6 (27.1) [c]	0.7	[60]
62	22-27	10842.0±3696.0 (45.4) [b]	9213.2 (41.8) [c]	0.8	58.6±30.5 (52.0) [b]	66.9 (27.1) [c]	1.1	[61]
63	23-43	12693.0±3243.0 (29.9) [b]	9213.2 (41.8) [c]	0.7	78.5±21.4 (27.3) [b]	66.9 (27.1) [c]	0.9	[62]

[a]Mean; [b]Mean±SD(CV%); [c]50th percentile of simulated AUC_{0-t}/C_{max}(CV%); [d]Mean(95% confidence interval); [e]Mean(CV%)

Continued Table S12

No.	age	AUC_{0-t} (ng/mL*min)			C_{max}(ng/mL)			Ref.
		Obs	Pre	Ratio	Obs	Pre	Ratio	
64	55-62	16569.0±8712.0 (68.6) [b]	9213.2 (41.8) [c]	0.6	98.0±27.3 (27.9) [b]	66.9 (27.1) [c]	0.7	[62]
65	22-37	18731.8 [a]	18266.3 (41.8) [c]	0.5	101.2 [a]	104.4 (27.1) [c]	1.0	[63]
66	32	16748.4 [a]	9219.0 (41.8) [c]	0.6	91.2 [a]	53.7 (27.1) [c]	0.6	[64]
67	32	14740.7 [a]	14380.9 (26.6) [c]	1.0	/	/	/	[64]
68	30±8	9692.2 [a]	8657.7 (26.4) [c]	0.9	/	/	/	[65]
69	71.3±6.6	13366.9 [a]	21851.3 (22.9) [c]	1.6	/	/	/	[66]
70	71.3±6.6	10397.3 [a]	12486.4 (37.6) [c]	1.2	30.4 [a]	64.1 (24.3) [c]	2.1	[66]
71	65-80	18731.8 [a]	18266.3 (37.5) [c]	1.0	101.2 [a]	104.4 (24.2) [c]	1.0	[63]
72	70	16748.4 [a]	9219.0 (38.0) [c]	0.6	91.2 [a]	53.7 (24.5) [c]	0.6	[64]
73	70	14740.7 [a]	14380.9 (24.0) [c]	1.0	/	/	/	[64]
74	69±5	4560.3 [a]	9188.3 (23.2) [c]	2.0	/	/	/	[65]
75	66-80	2820.0 (1860.0,3780.0) [d]	2445.7 (35.6) [c]	1.4	12.0 (9.0,15.0) [d]	14.8 (24.3) [c]	1.2	[67]
76	66-80	7800.0(6660.0,8940.0) [d]	12384.8 (23.5) [c]	1.8	/	/	/	[67]
77	65-71	8238.0±4398.0 (56.4) [b]	5635.2 (38.2) [c]	0.8	45.9 [a]	33.2 (24.8) [c]	0.7	[68]

[a]Mean; [b]Mean±SD(CV%); [c]50th percentile of simulated AUC_{0-t}/C_{max}(CV%); [d]Mean(95% confidence interval); [e]Mean(CV%)

Table S13 . Observed and predicted values of AUC_{0-t} of fentanyl

No.	age	AUC_{0-t} (ng/mL*min)			Ref.
		Obs	Pre	Ratio	
1	1-9	1552.7 [a]	1223.6 (21.2) [c]	0.8	[69]
2	3-10m	970.7 [a]	1942.8 (18.1) [c]	2.0	[69]
3	5.6±2.7	55.4 [a]	76.4 (21.0) [c]	1.4	[70]
4	GA 38w + PNA 1 d	4099.3 [a]	5081.9 (21.8) [c]	1.2	[71]
5	GA 40w + PNA 3 d	4632.8 [a]	5484.8 (22.2) [c]	1.2	[71]
6	GA 32w + PNA 10h	8124.1 [a]	9489.6 (21.9) [c]	1.2	[72]
7	PNA:2d	3263.5 [a]	2352.7 (22.1) [c]	1.4	[73]
8	PNA:2d	3843.1 [a]	4893.7 (21.9) [c]	0.8	[73]
9	33.4±8.52	61.2±54.0 (88.2) [b]	81.7 (23.8) [c]	1.3	[74]
10	22-29	511.0±37.0 (7.2) [b]	545.4 (24.2) [c]	1.1	[75]
11	22-29	458.0 [a]	500.1 (24.3) [c]	1.1	[75]
12	22-29	448.0 [a]	589.2 (24.3) [c]	1.3	[75]
13	22-29	445.0 [a]	461.2 (24.3) [c]	1.0	[75]
14	22-29	601.0 [a]	609.3 (24.3) [c]	1.0	[75]
15	32.7±8.8	264.0±90.0 (34.1) [b]	412.3 (24.4) [c]	1.6	[76]
16	53±17.6	233.0±70.0 (30.0) [b]	240.5 (24.0) [c]	1.0	[77]
17	18-41	784.6 [a]	1115.5 (18.8) [c]	1.4	[78]
18	55.67±8.22	1294.7 [a]	1716.9 (17.6) [c]	1.3	[79]
19	58±6	2424.0 [a]	2101.3 (33.3) [c]	0.9	[79]
20	56.6±7.5	1019.4 [a]	1963.2 (16.7) [c]	1.9	[79]
21	36±4	561.6 [a]	750.3 (24.2) [c]	1.3	[80]

[a]Mean; [b]Mean±SD(CV%); [c]50th percentile of simulated AUC_{0-t}/C_{max}(CV%); [d]Mean(95% confidence interval); [e]Mean(CV%)

Continued Table S13

No.	age	AUC$_{0-t}$ (ng/mL*min)			Ref.
		Obs	Pre	Ratio	
22	33	697.4 [a]	818.1 (24.2) [c]	1.2	[80]
23	27.8±3.8	1094.9 [a]	1208.5 (24.9) [c]	1.1	[82]
24	59±6	220.5 [a]	335.7 (18.5) [c]	1.5	[83]
25	23.86±1.81	82.7±23.3 (28.2) [b]	74.4 (19.1) [c]	0.9	[84]
26	58±11	2602.4 [a]	3034.9 (16.0) [c]	1.2	[85]
27	49±14	2533.0 [a]	3034.9 (16.0) [c]	1.2	[85]
28	55±12	3053.4 [a]	5255.0 (23.2) [c]	1.7	[85]
29	54±12	715.3 [a]	704.4 (23.2) [c]	1.0	[86]
30	44±15	1232.0 [a]	1408.8 (23.2) [c]	1.1	[86]
31	56±12	1974.7 [a]	2817.6 (23.2) [c]	1.4	[86]
32	54±12	2636.2 [a]	3522.0 (23.2) [c]	1.3	[86]
33	19-32	102.2±22.6 (22.1) [b]	117.3 (24.3) [c]	1.1	[87]
34	25.3±7.2	22.1±5.4 (24.4) [b]	27.1 (24.6) [c]	1.2	[88]
35	22.3±3.2	514.7 [a]	584.4 (24.2) [c]	1.1	[89]
36	22	583.0 [a]	584.4 (24.2) [c]	1.0	[89]
37	34	364.3 [a]	584.4 (24.2) [c]	1.6	[89]
38	35	440.2 [a]	584.4 (24.2) [c]	1.3	[89]
39	26	311.8 [a]	584.4 (24.2) [c]	1.9	[89]
40	23-31	1402.2 [a]	1284.7 (24.7) [c]	0.9	[90]
41	33-57	941.5 [a]	942.5 (23.9) [c]	1.0	[91]
42	30±5	362.4±131.4 (36.3) [b]	436.0 (24.1) [c]	1.2	[93]
43	49±12	6356.0 [a]	10257.1 (25.1) [c]	1.6	[92]
44	71-82	759.1 [a]	1017.9 (17.6) [c]	1.3	[78]
45	67±2	876.9 [a]	1073.4 (24.7) [c]	1.2	[80]
46	65	1047.9 [a]	1415.3 (13.6) [c]	1.4	[80]
47	61±8	4197.3 [a]	4000.9 (23.6) [c]	1.0	[85]
48	67.2±8.7	10551.8 [a]	19551.6 (20.8) [c]	1.9	[94]

[a]Mean; [b]Mean±SD(CV%); [c]50th percentile of simulated AUC$_{0-t}$/C$_{max}$(CV%); [d]Mean(95% confidence interval); [e]Mean(CV%)

Table S14 . Observed and predicted values of AUC_{0-t} of alfentanil

No.	age	AUC0-t (ng/mL*min)			Ref.
		Obs	Pre	Ratio	
1	4.97±0.85	2133.0 [a]	3824.2 (26.2) [c]	1.8	[95]
2	3.4±4.1	6069.2 [a]	5800.1 (29.0) [c]	1.0	[97]
3	GA 29.5±3.3w + PNA 1-3d	10092.8 [a]	6486.9 (17.7) [c]	0.6	[98]
4	5±2.8	6115.3 [a]	3446.7 (28.6) [c]	0.6	[98]
5	27-44	9217.3 [a]	10070.0 (29.2) [c]	1.1	[99]
6	43±9	18788.5 [a]	19326.3 (29.7) [c]	1.0	[100]
7	19-26	6680.0±3480.0 (52.1) [b]	4499.3 (26.2) [c]	0.8	[101]
8	51±6	324749.1 [a]	370253.3 (27.5) [c]	1.1	[92]
9	30±5	3264.0±1752.0 (53.7) [b]	3579.8 (27.5) [c]	1.2	[93]
10	38.8±6.79	21412.5 [a]	16674.9 (29.8) [c]	0.8	[102]
11	42±5.8	32525.9 [a]	33598.8 (26.2) [c]	1.0	[103]
12	45±9.8	8389.3 [a]	14218.6 (26.2) [c]	1.7	[103]
13	49±15	11430.5 [a]	9175.3 (26.2) [c]	0.8	[83]
14	68-91	14023.3 [a]	16802.3 (19.1) [c]	1.2	[99]

[a]Mean; [b]Mean±SD(CV%); [c]50th percentile of simulated AUC_{0-t}/C_{max}(CV%); [d]Mean(95% confidence interval); [e]Mean(CV%)

Table S15 . Observed and predicted values of AUC_{0-t} of sufentanil

No.	age	AUC0-t (ng/mL*min)			Ref.
		Obs	Pre	Ratio	
1	5.13±2.29	56.0 [a]	71.6 (19.5) [c]	1.3	[104]
2	5.42±1.06	98.0 [a]	107.0 (22.8) [c]	1.1	[104]
3	4.8±1.12	101.2 [a]	128.9 (22.1) [c]	1.3	[104]
4	45.5±13.73	409.3 [a]	581.2 (24.4) [c]	1.4	[105]
5	58±13.9	90.0±24.0 (26.7) [b]	103.1 (18.3) [c]	1.8	[106]
6	24-46	7.3 [a]	5.9 (20.4) [c]	0.8	[107]
7	41±15	104.5 [a]	97.7 (24.4) [c]	0.9	[108]
8	55.8±16.7	590.5 [a]	633.3 (24.4) [c]	0.9	[109]
9	77±5	106.3 [a]	167.2 (20.2) [c]	1.6	[108]
10	60.6±11.6	1401.0±454.0 (32.4) [b]	983.9 (18.8) [c]	0.7	[110]
11	61.5±10.2	1274.0±384.0 (30.1) [b]	1098.1 (19.3) [c]	0.7	[110]
12	61.3±7.8	1169.0±420.0 (35.9) [b]	1021.2 (19.2) [c]	1.3	[110]

[a]Mean; [b]Mean±SD(CV%); [c]50th percentile of simulated AUC_{0-t}/C_{max}(CV%); [d]Mean(95% confidence interval); [e]Mean(CV%)

Table S16 . The AFE and PE(%)of AUC_{0-t} and C_{max} in pediatrics、adults and geriatrics

Population	AFE	PE(%)
pediatrics	1.1	24.0
adults	0.9	15.1
geriatrics	1.1	24.2

3. List of Figures

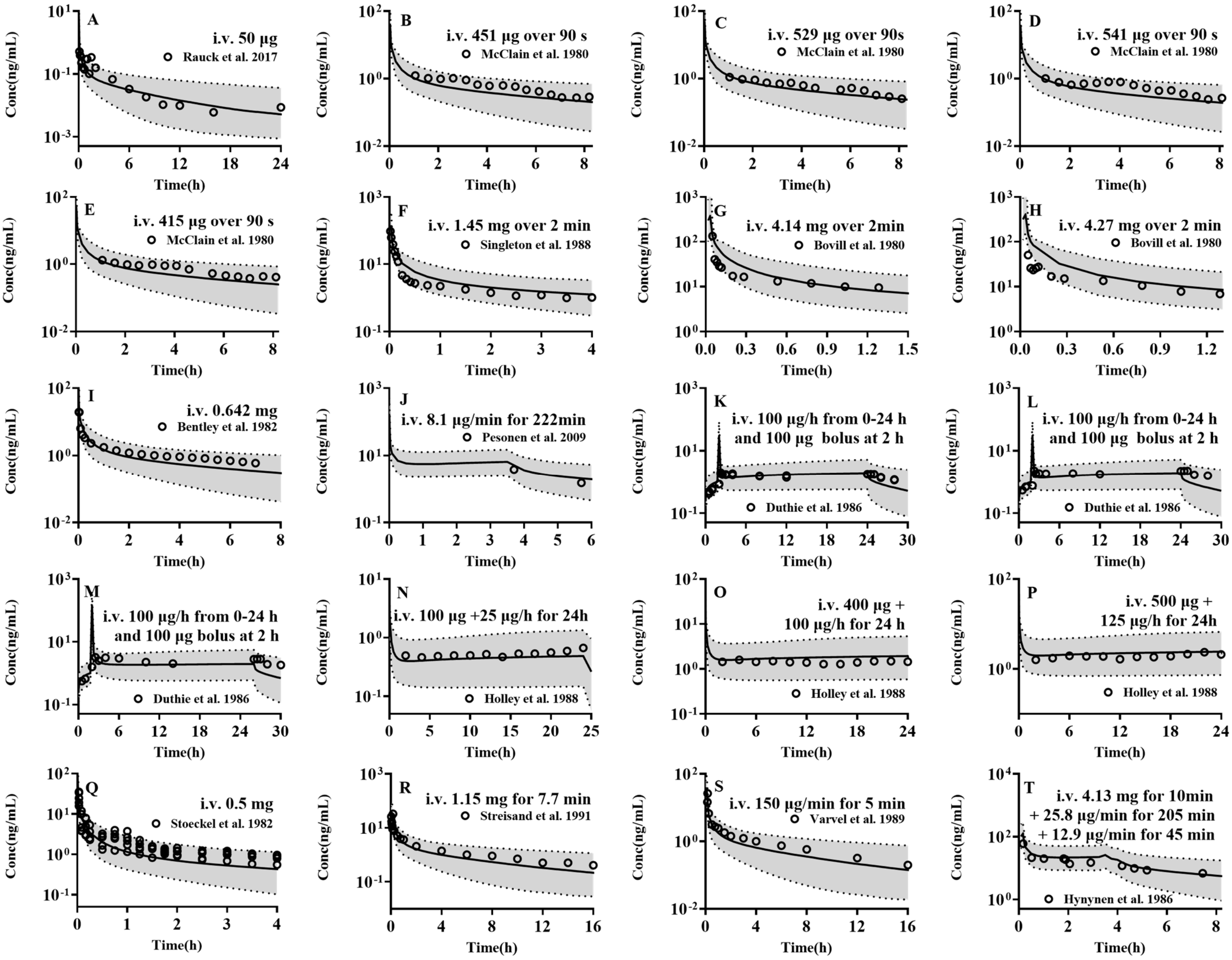

Figure S2. The predicted (lines) and observed (points) plasma concentrations of fentanyl following intravenous administration of fentanyl to adults. Solid line, 50th percentile of simulated plasma concentrations; Shadow, 5th-95th interval of the simulated plasma concentrations.

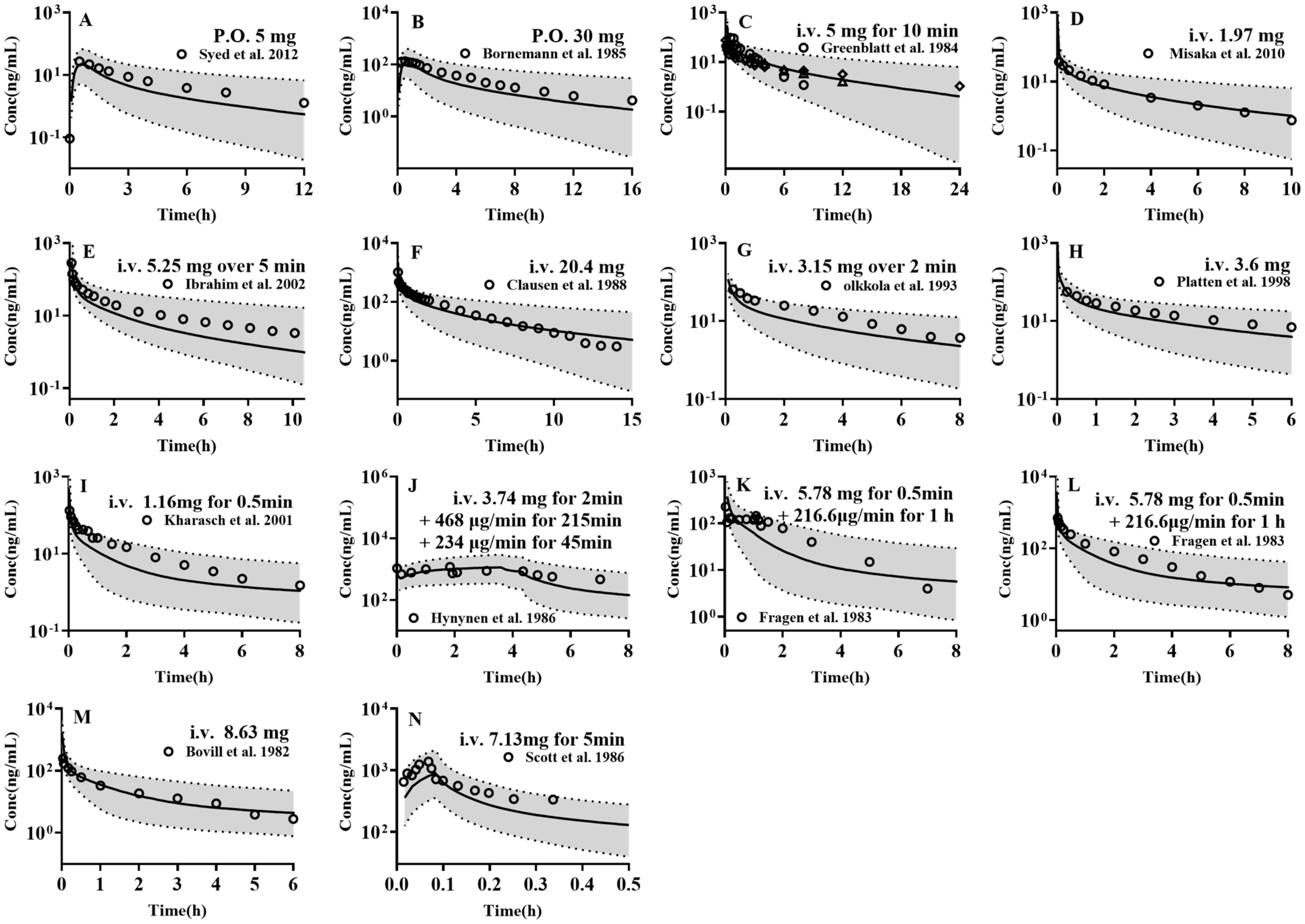

Figure S3. The predicted (lines) and observed (points) plasma concentrations of (**A-H**) midazolam, (**I-N**) alfentanil following oral and intravenous administration of midazolam or alfentanil to adults. Solid line, 50th percentile of simulated plasma concentrations; Shadow, 5th-95th interval of the simulated plasma concentration.

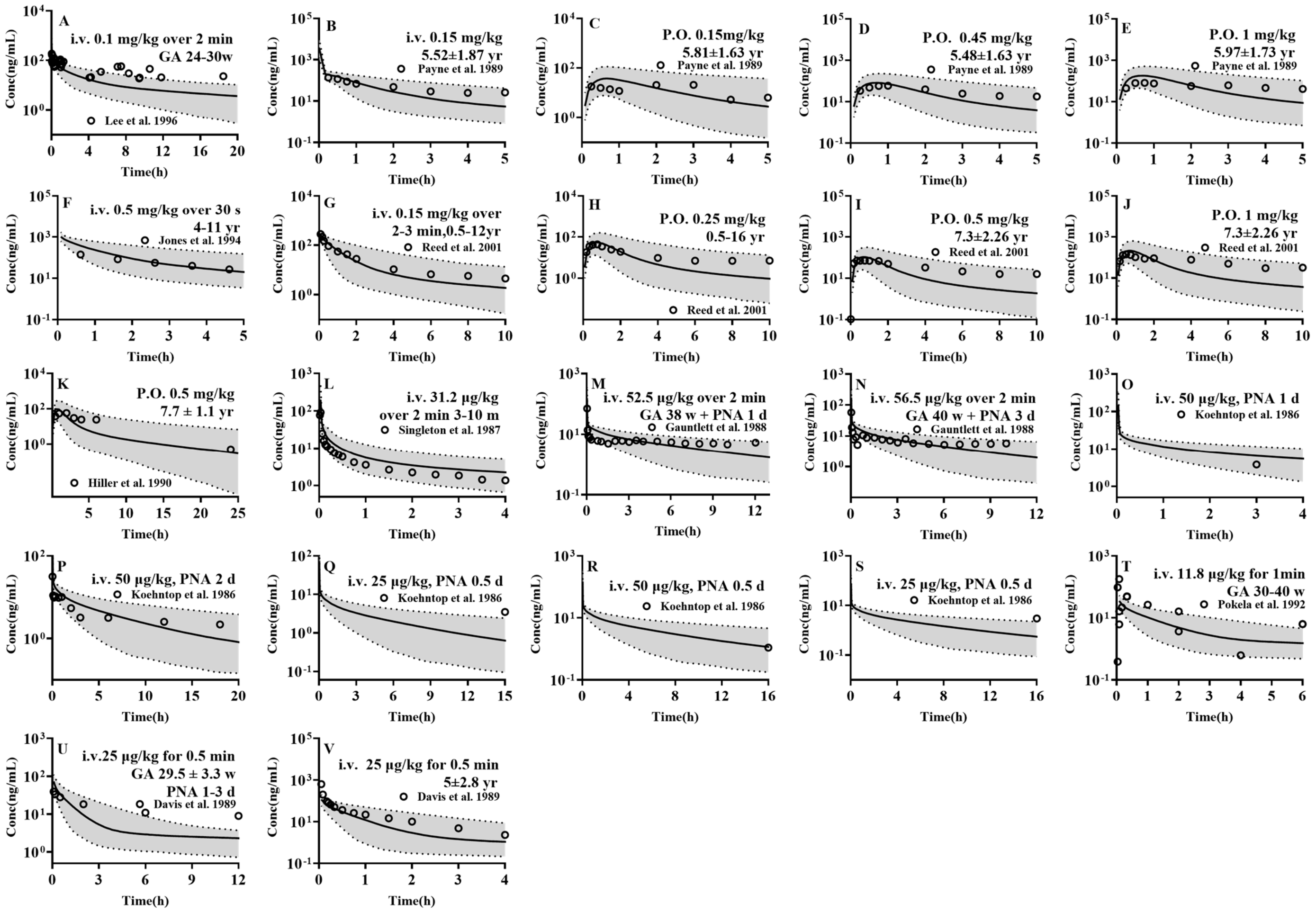

Figure S4. The predicted (lines) and observed (points) plasma concentrations of (**A-K**) midazolam, (**L-S**) fentanyl and (**T-V**) alfentanil following oral administration and intravenous administration of midazolam, fentanyl or alfentanil to pediatrics. Solid line, 50th percentile of simulated plasma concentrations; Shadow, 5th-95th interval of the simulated plasma concentrations.

4. Mode code of human following oral midazolam

```
test(){
deriv(Alungs = (Qtotal * Cvenous)- (Qtotal * Clungs/(Kplungs/Rb)))
deriv(Akidneys = - (Qkidneys * Ckidneys/(Kpkidneys/Rb)) + (Qkidneys * Cartery))
deriv(Aheart = - (Qheart * Cheart/(Kpheart/Rb)) + (Qheart * Cartery))
deriv(Aliver = - (Qliver+ Qstomach+ Qg1 + Qg2 + Qg3+ Qg4 + Qg5 +Qspleen) * Cliver/(Kpliver/Rb) + Qstomach * Cstomach/(Kpstomach/Rb) + (Cg1/(Kpintestines/Rb) * Qg1 + Cg2/(Kpintestines/Rb) * Qg2 + Cg3/(Kpintestines/Rb) * Qg3+Cg4 * Qg4/(Kpintestines/Rb)+Cg5 * Qg5/(Kpintestines/Rb)) + Qspleen * Cspleen/(Kpspleen/Rb) + Qliver * Cartery - PBSF * Cliver/(Kpliver/Rb) * fub *Clint)

stparm(Clint= tvClint*exp(nClint))
ranef(diag(nClint)= c(0.405465108))
fixef(tvClint(freeze) = c(,0.389,))

deriv(Amuscle = - (Qmuscle * Cmuscle/(Kpmuscle/Rb)) + (Qmuscle * Cartery))
deriv(Askin = - (Qskin * Cskin/(Kpskin/Rb)) + (Qskin * Cartery))
deriv(Aadipose = - (Qadipose * Cadipose/(Kpadipose/Rb)) + (Qadipose * Cartery))
deriv(Abrain = - (Qbrain * Cbrain/(Kpbrain/Rb)) + (Qbrain * Cartery))
deriv(Avenous = - Qtotal * Cvenous + Qkidneys * Ckidneys/(Kpkidneys/Rb) + Qheart * Cheart/(Kpheart/Rb) + Qbrain * Cbrain/(Kpbrain/Rb) + (Qliver+ Qstomach + Qg1 + Qg2 + Qg3 + Qg4 + Qg5 + Qspleen) * Cliver/(Kpliver/Rb) + Qmuscle * Cmuscle/(Kpmuscle/Rb) + Qskin * Cskin/(Kpskin/Rb) + Qadipose * Cadipose/(Kpadipose/Rb) + Qrob*Crob/(Kprob/Rb))

deriv(Aspleen = - (Qspleen * Cspleen/(Kpspleen/Rb)) + (Qspleen * Cartery))
deriv(Aartery = (Qtotal * Clungs/(Kplungs/Rb))-(Qtotal *Cartery))
deriv(A0 = - Kt0 * A0)
deriv(A1 = Kt0 * A0 - Kt1 * A1 - Ka1 * A1)
deriv(A2 = Kt1 * A1 - Kt2 * A2 - Ka2 * A2)
deriv(A3 = Kt2 * A2 - Kt3 * A3 - Ka3 * A3)
deriv(A4 = Kt3 * A3 - Kt4 * A4 )
deriv(A5 = Kt4 * A4 - Kt5 * A5 )
deriv(Astomach=Qstomach*Cartery-Qstomach*Cstomach/(Kpstomach/Rb))
deriv(Ag1 = Cartery * Qg1 + A1 * Ka1-Cg1/(Kpintestines/Rb)*fug*Clintg1 - Cg1 * Qg1/(Kpintestines/Rb))
deriv(Ag2 = Cartery * Qg2 + A2 * Ka2-Cg2/(Kpintestines/Rb)*fug*Clintg2- Cg2 * Qg2/(Kpintestines/Rb))
deriv(Ag3 = Cartery * Qg3 + A3 * Ka3 -Cg3/(Kpintestines/Rb)*fug*Clintg3- Cg3 * Qg3/(Kpintestines/Rb))
deriv(Ag4 = Cartery * Qg4 - Cg4 * Qg4/(Kpintestines/Rb))
deriv(Ag5 = Cartery * Qg5 - Cg5 * Qg5/(Kpintestines/Rb))

fug=1
stparm(Clintg1 = tvClintg1*exp(nClintg1))
ranef(diag(nClintg1) = c(0.405465108))
stparm(Clintg2 = tvClintg2*exp(nClintg2))
ranef(diag(nClintg2) = c(0.405465108))
stparm(Clintg3 = tvClintg3*exp(nClintg3))
ranef(diag(nClintg3) = c(0.405465108))
fixef(tvClintg1(freeze)= c(,34,))
fixef(tvClintg2(freeze)= c(,134.6,))
fixef(tvClintg3(freeze)= c(,78.51,))

Clungs = Alungs / Vlungs
Ckidneys = Akidneys/Vkidneys
Cheart = Aheart/Vheart
Cliver = Aliver/Vliver
Cmuscle = Amuscle/Vmuscle
Cskin = Askin/Vskin
```

```
Cadipose = Aadipose/Vadipose
Cbrain = Abrain/Vbrain
Cvenous = Avenous/Vvenous
Cspleen = Aspleen/Vspleen
Cartery = Aartery/Vartery

Cstomach=Astomach/Vstomach
Cg1 = Ag1/Vg1
Cg2 = Ag2/Vg2
Cg3 = Ag3/Vg3
Cg4 = Ag4/Vg4
Cg5 = Ag5/Vg5
Ka1 = 2*Peff1/r1
Ka2 = 2*Peff1/r2
Ka3 = 2*Peff1/r3

dosepoint(A0)
error(CEpsa=1)
observe(Cvenousobs=Cvenous/Rb+CEpsa)
Cpre=Cvenous/Rb

stparm(Vlungs = tvVlungs)
stparm(Vkidneys = tvVkidneys)
stparm(Vheart = tvVheart)
stparm(Vliver = tvVliver)
stparm(Vmuscle = tvVmuscle)
stparm(Vskin = tvVskin)
stparm(Vadipose = tvVadipose)
stparm(Vbrain = tvVbrain)
stparm(Vvenous = tvVvenous)
stparm(Vspleen = tvVspleen)
stparm(Vartery = tvVartery)

stparm(Qtotal = tvQtotal)
stparm(Qkidneys = tvQkidneys)
stparm(Qheart = tvQheart)
stparm(Qbrain = tvQbrain)
stparm(Qliver = tvQliver)
stparm(Qmuscle = tvQmuscle)
stparm(Qskin = tvQskin)
stparm(Qbrain = tvQbrain)
stparm(Qadipose = tvQadipose)
stparm(Qstomach = tvQstomach)
stparm(Qspleen = tvQspleen)

stparm(Kpadipose = tvKpadipose)
stparm(Kplungs = tvKplungs)
stparm(Kpliver = tvKpliver)
stparm(Kpstomach = tvKpstomach)
stparm(Kpintestines = tvKpintestines)
stparm(Kpbrain = tvKpbrain)
stparm(Kpkidneys = tvKpkidneys)
stparm(Kpheart=tvKpheart)
stparm(Kpmuscle = tvKpmuscle)
stparm(Kpskin = tvKpskin)
```

```
stparm(Kpspleen = tvKpspleen)

stparm(fub = tvfub*exp(nfub))
ranef(diag(nfub) = c(0.405465108))

stparm(PBSF=tvPBSF*exp(nPBSF))
ranef(diag(nPBSF) = c(0.405465108))
stparm(Rb=tvRb*exp(nRb))
ranef(diag(nRb) = c(0.405465108))

stparm(r1 = tvr1)
stparm(r2 = tvr2)
stparm(r3 = tvr3)

stparm(Peff1 = tvPeff1*exp(nPeff1))
ranef(diag(nPeff1) = c(0.405465108))

stparm(Kt0 = tvKt0)
stparm(Kt1 = tvKt1)
stparm(Kt2 = tvKt2)
stparm(Kt3 = tvKt3)
stparm(Kt4 = tvKt4)
stparm(Kt5 = tvKt5)

stparm(Vg1 = tvVg1)
stparm(Vg2 = tvVg2)
stparm(Vg3 = tvVg3)
stparm(Vg4 = tvVg4)
stparm(Vg5 = tvVg5)

stparm(Qg1 = tvQg1)
stparm(Qg2 = tvQg2)
stparm(Qg3 = tvQg3)
stparm(Qg4 = tvQg4)
stparm(Qg5 = tvQg5)

fixef(tvVlungs(freeze) = c(,1170,))
fixef(tvVkidneys(freeze) = c(,280,))
fixef(tvVheart(freeze) = c(,310,))
fixef(tvVliver(freeze) = c(,1690,))
fixef(tvVmuscle(freeze) = c(,35000,))
fixef(tvVskin(freeze) = c(,7800,))
fixef(tvVbrain(freeze) = c(,1450,))
fixef(tvVadipose(freeze) = c(,10000,))
fixef(tvVvenous(freeze) = c(,3470,))
fixef(tvVspleen(freeze) = c(,190,))
fixef(tvVartery(freeze) = c(,1730,))

fixef(tvQtotal(freeze) = c(,5600,))
fixef(tvQkidneys(freeze) = c(,1240,))
fixef(tvQheart(freeze) = c(,240,))
fixef(tvQbrain(freeze) = c(,700,))
fixef(tvQliver(freeze) = c(,300,))
fixef(tvQmuscle(freeze)    = c(,750,))
fixef(tvQskin(freeze) = c(,300,))
```

```
fixef(tvQadipose(freeze) = c(,260,))
fixef(tvQstomach (freeze) = c(,38.33,))
fixef(tvQspleen(freeze) = c(,80,))

fixef(tvKplungs(freeze) = c(,2.506,))
fixef(tvKpmuscle(freeze) = c(,0.724,))
fixef(tvKpheart(freeze) = c(,2.339,))
fixef(tvKpbrain(freeze) = c(,1.838,))
fixef(tvKpadipose(freeze) = c(,5.013,))
fixef(tvKpskin(freeze) = c(,0.780,))
fixef(tvKpintestines (freeze) = c(,2.673,))
fixef(tvKprob(freeze)=c(,0.001,))
fixef(tvKpliver(freeze) = c(,2.228,))
fixef(tvKpkidneys(freeze) = c(,2.562,))
fixef(tvKpspleen(freeze) = c(,1.838,))
fixef(tvKpstomach (freeze) = c(,4.233,))

fixef(tvfub(freeze)=c(,0.044,))
fixef(tvPBSF(freeze)=c(,64220,))
fixef(tvRb(freeze)=c(,0.6,))
fixef(tvKt0(freeze) = c(,0.08,))
fixef(tvKt1(freeze) = c(,0.07,))
fixef(tvKt2(freeze) = c(,0.03,))
fixef(tvKt3(freeze) = c(,0.04,))
fixef(tvKt4(freeze) = c(,0.003,))
fixef(tvKt5(freeze) = c(,0.001,))

fixef(tvVg1(freeze) = c(,70,))
fixef(tvVg2(freeze) = c(,209,))
fixef(tvVg3(freeze) = c(,139,))
fixef(tvVg4(freeze) = c(,116,))
fixef(tvVg5(freeze) = c(,1116,))

fixef(tvQg1(freeze) = c(,118,))
fixef(tvQg2(freeze) = c(,413,))
fixef(tvQg3(freeze) = c(,244,))
fixef(tvQg4(freeze) = c(,44,))
fixef(tvQg5(freeze) = c(,281,))

fixef(tvr1(freeze) = c(,2,))
fixef(tvr2(freeze) = c(,1.63,))
fixef(tvr3(freeze) = c(,1.45,))
fixef(tvPeff1(freeze) = c(,0.0264,))

stparm(Vstomach = tvVstomach)
fixef(tvVstomach(freeze) = c(,160,))

deriv(Arob = - (Qrob * Crob/(Kprob/Rb)) + (Qrob * Cartery))
Crob=Arob/Vrob
stparm(Vrob=tvVrob)
stparm(Qrob=tvQrob)
stparm(Kprob=tvKprob)
fixef(tvVrob(freeze)=c(,5100,))
fixef(tvQrob(freeze)=c(,592,))
}
```

Reference

1. Gertz, M.; Houston, J.B.; Galetin, A. Physiologically based pharmacokinetic modeling of intestinal first-pass metabolism of CYP3A substrates with high intestinal extraction. *Drug Metab Dispos* **2011**, *39*, 1633-1642, doi:10.1124/dmd.111.039248.
2. Yu, L.X.; Amidon, G.L. A compartmental absorption and transit model for estimating oral drug absorption. *Int J Pharm* **1999**, *186*, 119-125, doi:10.1016/s0378-5173(99)00147-7.
3. Guo, H.; Liu, C.; Li, J.; Zhang, M.; Hu, M.; Xu, P.; Liu, L.; Liu, X. A mechanistic physiologically based pharmacokinetic-enzyme turnover model involving both intestine and liver to predict CYP3A induction-mediated drug–drug interactions. *Journal of pharmaceutical sciences* **2013**, *102*, 2819-2836.
4. Rostami-Hodjegan, A.; Tucker, G.T. Simulation and prediction of in vivo drug metabolism in human populations from in vitro data. *Nature reviews Drug discovery* **2007**, *6*, 140-148.
5. Yang, J.; Jamei, M.; Yeo, K.R.; Tucker, G.T.; Rostami-Hodjegan, A. Prediction of intestinal first-pass drug metabolism. *Current drug metabolism* **2007**, *8*, 676-684.
6. Yeo, K.R.; Jamei, M.; Yang, J.; Tucker, G.T.; Rostami-Hodjegan, A. Physiologically based mechanistic modelling to predict complex drug–drug interactions involving simultaneous competitive and time-dependent enzyme inhibition by parent compound and its metabolite in both liver and gut—the effect of diltiazem on the time-course of exposure to triazolam. *European Journal of Pharmaceutical Sciences* **2010**, *39*, 298-309.
7. Brun, J.; Bouchahda, C.; Chaze, D.; Aïssa Benhaddad, A.; Micallef, J.; Mercier, J. The paradox of hematocrit in exercise physiology: which is the “normal” range from an hemorheologist's viewpoint? *Clinical hemorheology and microcirculation* **2000**, *22*, 287-303.
8. Jeon, S.R.; Park, J.H.; Ahn, Y. Fentanyl: General Properties and Therapeutic Uses.
9. Lüllmann, H.; MARTINS, B.-S.; Peters, T. pH-dependent accumulation of fentanyl, lofentanil and alfentanil by beating guineapig atria. *British journal of anaesthesia* **1985**, *57*, 1012-1017.
10. Metz, C.; Göbel, L.; Gruber, M.; Hoerauf, K.H.; Taeger, K. Pharmacokinetics of human cerebral opioid extraction: a comparative study on sufentanil, fentanyl, and alfentanil in a patient after severe head injury. *The Journal of the American Society of Anesthesiologists* **2000**, *92*, 1559-1567.
11. Zhou, W.; Johnson, T.N.; Bui, K.H.; Cheung, S.A.; Li, J.; Xu, H.; Al-Huniti, N.; Zhou, D. Predictive performance of physiologically based pharmacokinetic (PBPK) modeling of drugs extensively metabolized by major cytochrome P450s in children. *Clinical Pharmacology & Therapeutics* **2018**, *104*, 188-200.
12. Shum, S.; Shen, D.D.; Isoherranen, N. Predicting maternal-fetal disposition of fentanyl following intravenous and epidural administration using physiologically based pharmacokinetic modeling. *Drug Metabolism and Disposition* **2021**, *49*, 1003-1015.
13. Baneyx, G.; Parrott, N.; Meille, C.; Iliadis, A.; Lavé, T. Physiologically based pharmacokinetic modeling of CYP3A4 induction by rifampicin in human: influence of time between substrate and inducer administration. *European Journal of Pharmaceutical Sciences* **2014**, *56*, 1-15.
14. Zhu, J.; Zhao, Y.; Wang, L.; Zhou, C.; Zhou, S.; Chen, T.; Chen, J.; Zhang, Z.; Zhu, Y.; Ding, S. Physiologically based pharmacokinetic/pharmacodynamic modeling to evaluate the absorption of midazolam rectal gel. *European Journal of Pharmaceutical Sciences* **2021**, *167*, 106006.
15. BOWER, S. Plasma protein binding of fentanyl. *Journal of Pharmacy and Pharmacology* **1981**, *33*, 507-514.
16. Gertz, M.; Harrison, A.; Houston, J.B.; Galetin, A. Prediction of human intestinal first-pass metabolism of 25 CYP3A substrates from in vitro clearance and permeability data. *Drug Metabolism and Disposition* **2010**, *38*, 1147-1158.
17. Nishimuta, H.; Sato, K.; Yabuki, M.; Komuro, S. Prediction of the intestinal first-pass metabolism of CYP3A and UGT substrates in humans from in vitro data. *Drug metabolism and pharmacokinetics* **2011**, *26*, 592-601.
18. Encinas, E.; Calvo, R.; Lukas, J.C.; Vozmediano, V.; Rodriguez, M.; Suarez, E. A predictive pharmacokinetic/pharmacodynamic model of fentanyl for analgesia/sedation in neonates based on a semi-physiologic approach. *Pediatric drugs* **2013**, *15*, 247-257.
19. Kazmi, F. System-dependent metabolism of drugs by cytochrome p450: the mechanistic basis for why human liver microsomes are superior to human hepatocytes at metabolizing midazolam but inferior at metabolizing desloratadine. University of Kansas, 2015.
20. Scholz, J.; Bause, H.; Schulz, M.; Klotz, U.; Krishna, D.; Pohl, S.; Schulte am Esch, J. Pharmacokinetics and effects on intracranial pressure of sufentanil in head trauma patients. *British journal of clinical pharmacology* **1994**, *38*, 369-372.

21. Rodgers, T.; Leahy, D.; Rowland, M. Physiologically based pharmacokinetic modeling 1: predicting the tissue distribution of moderate-to-strong bases. *Journal of pharmaceutical sciences* **2005**, *94*, 1259-1276.
22. Björkman, S.; Fyge, Å.; Qi, Z. Determination of the steady state tissue distribution of midazolam in the rat. *Journal of pharmaceutical sciences* **1996**, *85*, 887-889.
23. Kong, W.-m.; Sun, B.-b.; Wang, Z.-j.; Zheng, X.-k.; Zhao, K.-j.; Chen, Y.; Zhang, J.-x.; Liu, P.-h.; Zhu, L.; Xu, R.-j. Physiologically based pharmacokinetic–pharmacodynamic modeling for prediction of vonoprazan pharmacokinetics and its inhibition on gastric acid secretion following intravenous/oral administration to rats, dogs and humans. *Acta Pharmacologica Sinica* **2020**, *41*, 852-865.
24. Hakooz, N.; Ito, K.; Rawden, H.; Gill, H.; Lemmers, L.; Boobis, A.R.; Edwards, R.J.; Carlile, D.J.; Lake, B.G.; Houston, J.B. Determination of a human hepatic microsomal scaling factor for predicting in vivo drug clearance. *Pharmaceutical research* **2006**, *23*, 533-539.
25. Barter, Z.E.; Bayliss, M.K.; Beaune, P.H.; Boobis, A.R.; Carlile, D.J.; Edwards, R.J.; Brian Houston, J.; Lake, B.G.; Lipscomb, J.C.; Pelkonen, O.R. Scaling factors for the extrapolation of in vivo metabolic drug clearance from in vitro data: reaching a consensus on values of human micro-somal protein and hepatocellularity per gram of liver. *Current drug metabolism* **2007**, *8*, 33-45.
26. Price, K.; Haddad, S.; Krishnan, K. Physiological modeling of age-specific changes in the pharmacokinetics of organic chemicals in children. *Journal of Toxicology and Environmental Health Part A* **2003**, *66*, 417-433.
27. Haddad, S.; Restieri, C.; Krishnan, K. Characterization of age-related changes in body weight and organ weights from birth to adolescence in humans. *Journal of toxicology and environmental health Part A* **2001**, *64*, 453-464.
28. Björkman, S. Prediction of drug disposition in infants and children by means of physiologically based pharmacokinetic (PBPK) modelling: theophylline and midazolam as model drugs. *British journal of clinical pharmacology* **2005**, *59*, 691-704.
29. Chang, H.P.; Kim, S.J.; Wu, D.; Shah, K.; Shah, D.K. Age-related changes in pediatric physiology: quantitative analysis of organ weights and blood flows: age-related changes in pediatric physiology. *The AAPS Journal* **2021**, *23*, 1-15.
30. Stader, F.; Siccardi, M.; Battegay, M.; Kinvig, H.; Penny, M.A.; Marzolini, C. Repository describing an aging population to inform physiologically based pharmacokinetic models considering anatomical, physiological, and biological age-dependent changes. *Clinical pharmacokinetics* **2019**, *58*, 483-501.
31. Lee, T.C.; Charles, B. Measurement by HPLC of Midazolam and its Major Metabolite 1-Hydroxymidazolam in Plasma of Very Premature Neonates. *Biomedical Chromatography* **1996**, *10*, 65-68.
32. Mulla, H.; McCormack, P.; Lawson, G.; Firmin, R.K.; Upton, D.R. Pharmacokinetics of midazolam in neonates undergoing extracorporeal membrane oxygenation. *The Journal of the American Society of Anesthesiologists* **2003**, *99*, 275-282.
33. Malinovsky, J.-M.; Lejus, C.; Servin, F.; Lepage, J.-Y.; Normand, Y.L.; Testa, S.; Cozian, A.; Pinaud, M. Plasma concentrations of midazolam after iv, nasal or rectal administration in children. *BJA: British Journal of Anaesthesia* **1993**, *70*, 617-620.
34. Walbergh, E.J.; Wills, R.J.; Eckhert, J. Plasma concentrations of midazolam in children following intranasal administration. *Anesthesiology* **1991**, *74*, 233-235.
35. Rey, E.; Delaunay, L.; Pons, G.; Murat, I.; Richard, M.; Saint-Maurice, C.; Olive, G. Pharmacokinetics of midazolam in children: comparative study of intranasal and intravenous administration. *European journal of clinical pharmacology* **1991**, *41*, 355-357.
36. Salonen, M.; Kanto, J.; Himberg, J.-J. Midazolam as an induction agent in children: a pharmacokinetic and clinical study. *Anesthesia & Analgesia* **1987**, *66*, 625-628.
37. Tolia, V.; Brennan, S.; Aravind, M.; Kauffman, R.E. Pharmacokinetic and pharmacodynamic study of midazolam in children during esophagogastroduodenoscopy. *The Journal of pediatrics* **1991**, *119*, 467-471.
38. Payne, K.; Mattheyse, F.; Liebenberg, D.; Dawes, T. The pharmacokinetics of midazolam in paediatric patients. *European journal of clinical pharmacology* **1989**, *37*, 267-272.
39. Jones, R.; Visram, A.; Chan, M.; Bacon-Shone, J.; Mya, G.; Irwin, M. A comparison of three induction agents in paediatric anaesthesia—cardiovascular effects and recovery. *Anaesthesia and intensive care* **1994**, *22*, 545-555.
40. Reed, M.D.; Rodarte, A.; Blumer, J.L.; Khoo, K.C.; Akbari, B.; Pou, S.; Kearns, G.L. The single-dose pharmacokinetics of midazolam and its primary metabolite in pediatric patients after oral and intravenous administration. *The journal of Clinical pharmacology* **2001**, *41*, 1359-1369.
41. Hiller, A.; Olkkola, K.; Isohanni, P.; Saarnivaara, L. Unconsciousness associated with midazolam and erythromycin.

BJA: British Journal of Anaesthesia **1990**, *65*, 826-828.
42. Brosius, K.K.; Bannister, C.F. Midazolam premedication in children: a comparison of two oral dosage formulations on sedation score and plasma midazolam levels. *Anesthesia & Analgesia* **2003**, *96*, 392-395.
43. Yang, G.; Fu, Z.; Chen, X.; Yuan, H.; Yang, H.; Huang, Y.; Ouyang, D.; Tan, Z.; Tan, H.; Huang, Z. Effects of the CYP oxidoreductase Ala503Val polymorphism on CYP3A activity in vivo: a randomized, open-label, crossover study in healthy Chinese men. *Clinical therapeutics* **2011**, *33*, 2060-2070.
44. Misaka, S.; Uchida, S.; Imai, H.; Inui, N.; Nishio, S.; Ohashi, K.; Watanabe, H.; Yamada, S. Pharmacokinetics and pharmacodynamics of low doses of midazolam administered intravenously and orally to healthy volunteers. *Clinical and Experimental Pharmacology and Physiology* **2010**, *37*, 290-295.
45. Hase, I.; Oda, Y.; Tanaka, K.; Mizutani, K.; Nakamoto, T.; Asada, A. Iv fentanyl decreases the clearance of midazolam. *British journal of anaesthesia* **1997**, *79*, 740-743.
46. Yan, D.; Yang, Y.; Uchida, S.; Misaka, S.; Luo, J.; Takeuchi, K.; Inui, N.; Yamada, S.; Ohashi, K.; Watanabe, H. Effects of ursodeoxycholic acid on the pharmacokinetics and pharmacodynamics of intravenous and oral midazolam in healthy volunteers. *Naunyn-Schmiedeberg's archives of pharmacology* **2008**, *377*, 629-636.
47. Wermeling, D.P.; Record, K.A.; Archer, S.M.; Rudy, A.C. A pharmacokinetic and pharmacodynamic study, in healthy volunteers, of a rapidly absorbed intranasal midazolam formulation. *Epilepsy research* **2009**, *83*, 124-132.
48. Wermeling, D.P.; Record, K.A.; Kelly, T.H.; Archer, S.M.; Clinch, T.; Rudy, A.C. Pharmacokinetics and pharmacodynamics of a new intranasal midazolam formulation in healthy volunteers. *Anesthesia & Analgesia* **2006**, *103*, 344-349.
49. Ibrahim, A.; Karim, A.; Feldman, J.; Kharasch, E. The influence of parecoxib, a parenteral cyclooxygenase-2 specific inhibitor, on the pharmacokinetics and clinical effects of midazolam. *Anesthesia & Analgesia* **2002**, *95*, 667-673.
50. Syed, S.; Clemens, P.L.; Lathers, D.; Kollia, G.; Dhar, A.; Walters, I.; Masson, E. Lack of effect of brivanib on the pharmacokinetics of midazolam, a CYP3A4 substrate, administered intravenously and orally in healthy participants. *The Journal of Clinical Pharmacology* **2012**, *52*, 914-921.
51. Clausen, T.; Wolff, J.; Hansen, P.; Larsen, F.; Rasmussen, S.; Dixon, J.; Crevoisier, C. Pharmacokinetics of midazolam and alpha - hydroxy - midazolam following rectal and intravenous administration. *British journal of clinical pharmacology* **1988**, *25*, 457-463.
52. Shao, F.; Zhang, H.; Xie, L.; Chen, J.; Zhou, S.; Zhang, J.; Lv, J.; Hao, W.; Ma, Y.; Liu, Y. Pharmacokinetics of ginkgolides A, B and K after single and multiple intravenous infusions and their interactions with midazolam in healthy Chinese male subjects. *European journal of clinical pharmacology* **2017**, *73*, 537-546.
53. Bornemann, L.; Min, B.; Crews, T.; Rees, M.; Blumenthal, H.; Colburn, W.; Patel, I. Dose dependent pharmacokinetics of midazolam. *European journal of clinical pharmacology* **1985**, *29*, 91-95.
54. Greenblatt, D.J.; Peters, D.E.; Oleson, L.E.; Harmatz, J.S.; MacNab, M.W.; Berkowitz, N.; Zinny, M.A.; Court, M.H. Inhibition of oral midazolam clearance by boosting doses of ritonavir, and by 4, 4 - dimethyl - benziso - (2H) - selenazine (ALT - 2074), an experimental catalytic mimic of glutathione oxidase. *British journal of clinical pharmacology* **2009**, *68*, 920-927.
55. Olkkola, K.T.; Aranko, K.; Luurila, H.; Hiller, A.; Saarnivaara, L.; Himberg, J.J.; Neuvonen, P.J. A potentially hazardous interaction between erythromycin and midazolam. *Clinical Pharmacology & Therapeutics* **1993**, *53*, 298-305.
56. Olkkola, K.T.; Ahonen, J.; Neuvonen, P.J. The effect of the systemic antimycotics, itraconazole and fluconazole, on the pharmacokinetics and pharmacodynamics of intravenous and oral midazolam. *Anesthesia & Analgesia* **1996**, *82*, 511-516.
57. Palkama, V.J.; Ahonen, J.; Neuvonen, P.J.; Olkkola, K.T. Effect of saquinavir on the pharmacokinetics and pharmacodynamics of oral and intravenous midazolam. *Clinical Pharmacology & Therapeutics* **1999**, *66*, 33-39.
58. Saari, T.I.; Laine, K.; Leino, K.; Valtonen, M.; Neuvonen, P.J.; Olkkola, K.T. Effect of voriconazole on the pharmacokinetics and pharmacodynamics of intravenous and oral midazolam. *Clinical Pharmacology & Therapeutics* **2006**, *79*, 362-370.
59. Link, B.; Haschke, M.; Grignaschi, N.; Bodmer, M.; Aschmann, Y.Z.; Wenk, M.; Krähenbühl, S. Pharmacokinetics of intravenous and oral midazolam in plasma and saliva in humans: usefulness of saliva as matrix for CYP3A phenotyping. *British journal of clinical pharmacology* **2008**, *66*, 473-484.
60. Smith, M.; Eadie, M.; Brophy, T.O.R. The pharmacokinetics of midazolam in man. *European journal of clinical pharmacology* **1981**, *19*, 271-278.
61. Wang, J.-S.; Backman, J.T.; Kivistö, K.T.; Neuvonen, P.J. Effects of metronidazole on midazolam metabolism in vitro

and in vivo. *European journal of clinical pharmacology* **2000**, *56*, 555-559.
62. Wang, H.-y.; Chen, X.; Jiang, J.; Shi, J.; Hu, P. Evaluating a physiologically based pharmacokinetic model for predicting the pharmacokinetics of midazolam in Chinese after oral administration. *Acta Pharmacologica Sinica* **2016**, *37*, 276-284.
63. Castleden, C.; Allen, J.; Altman, J.; John-Smith, P.S. A comparison of oral midazolam, nitrazepam and placebo in young and elderly subjects. *European journal of clinical pharmacology* **1987**, *32*, 253-257.
64. Greenblatt, D.J.; Abernethy, D.R.; Locniskar, A.; Harmatz, J.S.; Limjuco, R.A.; Shader, R.I. Effect of age, gender, and obesity on midazolam kinetics. *Anesthesiology* **1984**, *61*, 27-35.
65. Platten, H.P.; Schweizer, E.; Dilger, K.; Mikus, G.; Klotz, U. Pharmacokinetics and the pharmacodynamic action of midazolam in young and elderly patients undergoing tooth extraction. *Clinical Pharmacology & Therapeutics* **1998**, *63*, 552-560.
66. Smith, M.; Heazlewood, V.; Eadie, M.; Brophy, T.O.r.; Tyrer, J. Pharmacokinetics of midazolam in the aged. *European journal of clinical pharmacology* **1984**, *26*, 381-388.
67. Quinney, S.K.; Haehner, B.D.; Rhoades, M.B.; Lin, Z.; Gorski, J.C.; Hall, S.D. Interaction between midazolam and clarithromycin in the elderly. *British journal of clinical pharmacology* **2008**, *65*, 98-109.
68. Krupka, E.; Venisse, N.; Lafay, C.; Gendre, D.; Diquet, B.; Bouquet, S.; Perault, M.-C. Probe of CYP3A by a single-point blood measurement after oral administration of midazolam in healthy elderly volunteers. *European journal of clinical pharmacology* **2006**, *62*, 653-659.
69. Singleton, M.A.; Rosen, J.I.; Fisher, D.M. Plasma concentrations of fentanyl in infants, children and adults. *Canadian journal of anaesthesia* **1987**, *34*, 152-155.
70. Dsida, R.M.; Wheeler, M.; Birmingham, P.K.; Henthorn, T.K.; Avram, M.J.; Enders-Klein, C.; Maddalozzo, J.; Cote, C.J. Premedication of pediatric tonsillectomy patients with oral transmucosal fentanyl citrate. *Anesthesia & Analgesia* **1998**, *86*, 66-70.
71. Gauntlett, I.S.; Fisher, D.M.; Hertzka, R.E.; Kuhls, E.; Spellman, M.J.; Rudolph, C. Pharmacokinetics of fentanyl in neonatal humans and lambs: effects of age. *Anesthesiology* **1988**, *69*, 683-687.
72. Saarenmaa, E.; Neuvonen, P.J.; Fellman, V. Gestational age and birth weight effects on plasma clearance of fentanyl in newborn infants. *The Journal of pediatrics* **2000**, *136*, 767-770.
73. Koehntop, D.E.; Rodman, J.H.; Brundage, D.M.; Hegland, M.G.; Buckley, J.J. Pharmacokinetics of fentanyl in neonates. *Anesthesia & Analgesia* **1986**, *65*, 227-232.
74. Rauck, R.; Oh, D.A.; Parikh, N.; Koch, C.; Singla, N.; Yu, J.; Nalamachu, S.; Vetticaden, S. Pharmacokinetics and safety of fentanyl sublingual spray and fentanyl citrate intravenous: a single ascending dose study in opioid-naïve healthy volunteers. *Current Medical Research and Opinion* **2017**, *33*, 1915-1920.
75. McClain, D.A.; Hug Jr, C.C. Intravenous fentanyl kinetics. *Clinical Pharmacology & Therapeutics* **1980**, *28*, 106-114.
76. Ziesenitz, V.C.; König, S.K.; Mahlke, N.S.; Skopp, G.; Haefeli, W.E.; Mikus, G. Pharmacokinetic interaction of intravenous fentanyl with ketoconazole. *The Journal of Clinical Pharmacology* **2015**, *55*, 708-717.
77. Nozari, A.; Akeju, O.; Mirzakhani, H.; Eskandar, E.; Ma, Z.; Hossain, M.A.; Wang, Q.; Greenblatt, D.J.; Martyn, J.J. Prolonged therapy with the anticonvulsant carbamazepine leads to increased plasma clearance of fentanyl. *Journal of Pharmacy and Pharmacology* **2019**, *71*, 982-987.
78. Singleton, M.; Rosen, J.; Fisher, D. Pharmacokinetics of fentanyl in the elderly. *British journal of anaesthesia* **1988**, *60*, 619-622.
79. Bovill, J.; Sebel, P. Pharmacokinetics of high-dose fentanyl: a study in patients undergoing cardiac surgery. *British journal of anaesthesia* **1980**, *52*, 795-801.
80. Bentley, J.B.; Borel, J.D.; Nenad Jr, R.E.; Gillespie, T.J. Age and fentanyl pharmacokinetics. *Anesthesia & Analgesia* **1982**, *61*, 968-971.
81. Pesonen, A.; SUOJARANTA-YLINEN, R.; Hammaren, E.; Tarkkila, P.; Seppälä, T.; Rosenberg, P.H. Comparison of effects and plasma concentrations of opioids between elderly and middle-aged patients after cardiac surgery. *Acta anaesthesiologica scandinavica* **2009**, *53*, 101-108.
82. Egan, T.D.; Sharma, A.; Ashburn, M.A.; Kievit, J.; Pace, N.L.; Streisand, J.B. Multiple dose pharmacokinetics of oral transmucosal fentanyl citrate in healthy volunteers. *The Journal of the American Society of Anesthesiologists* **2000**, *92*, 665-673.
83. Scott, J.C.; Ponganis, K.V.; Stanski, D.R. EEG quantitation of narcotic effect: the comparative pharmacodynamics of fentanyl and alfentanil. *Anesthesiology* **1985**, *62*, 234-241.

84. Christrup, L.L.; Foster, D.; Popper, L.D.; Troen, T.; Upton, R. Pharmacokinetics, efficacy, and tolerability of fentanyl following intranasal versus intravenous administration in adults undergoing third-molar extraction: a randomized, double-blind, double-dummy, two-way, crossover study. *Clinical therapeutics* **2008**, *30*, 469-481.
85. Duthie, D.; McLaren, A.; Nimmo, W. Pharmacokinetics of fentanyl during constant rate iv infusion for the relief of pain after surgery. *BJA: British Journal of Anaesthesia* **1986**, *58*, 950-956.
86. Holley, F.; Van Steennis, C. Postoperative analgesia with fentanyl: pharmacokinetics and pharmacodynamics of constant-rate iv and transdermal delivery. *BJA: British Journal of Anaesthesia* **1988**, *60*, 608-613.
87. Lim, C.B.S.; Schug, S.A.; Sunderland, V.B.; Paech, M.J.; Liu, Y. A phase I pharmacokinetic and bioavailability study of a sublingual fentanyl wafer in healthy volunteers. *Anesthesia & Analgesia* **2012**, *115*, 554-559.
88. MacLeod, D.B.; Habib, A.S.; Ikeda, K.; Spyker, D.A.; Cassella, J.V.; Ho, K.Y.; Gan, T.J. Inhaled fentanyl aerosol in healthy volunteers: pharmacokinetics and pharmacodynamics. *Anesthesia & Analgesia* **2012**, *115*, 1071-1077.
89. Stoeckel, H.; Schüttler, J.; Magnussen, H.; Hengstmann, J. Plasma fentanyl concentrations and the occurrence of respiratory depression in volunteers. *British Journal of Anaesthesia* **1982**, *54*, 1087-1095.
90. Streisand, J.B.; Varvel, J.R.; Stanski, D.R.; Le Maire, L.; Ashburn, M.A.; Hague, B.I.; Tarver, S.D.; Stanley, T.H. Absorption and bioavailability of oral transmucosal fentanyl citrate. *Anesthesiology* **1991**, *75*, 223-229.
91. Varvel, J.; Shafer, S.; Hwang, S.; Coen, P.; Stanski, D. Absorption characteristics of transdermally administered fentanyl. *Anesthesiology* **1989**, *70*, 928-934.
92. Hynynen, M.; Takkunen, O.; Salmenperä, M.; Haataja, H.; Heinonen, J. Continuous infusion of fentanyl or alfentanil for coronary artery surgery: plasma opiate concentrations, haemodynamics and postoperative course. *BJA: British Journal of Anaesthesia* **1986**, *58*, 1252-1259.
93. Ibrahim, A.E.; Feldman, J.; Karim, A.; Kharasch, E.D. Simultaneous assessment of drug interactions with low-and high-extraction opioids: application to parecoxib effects on the pharmacokinetics and pharmacodynamics of fentanyl and alfentanil. *The Journal of the American Society of Anesthesiologists* **2003**, *98*, 853-861.
94. Hudson, R.J.; Thomson, I.; Cannon, J.E.; Friesen, R.M.; Meatherall, R.C. Pharmacokinetics of fentanyl in patients undergoing abdominal aortic surgery. *Anesthesiology* **1986**, *64*, 334-338.
95. Roure, P.; Jean, N.; Leclerc, A.-C.; Cabanel, N.; Levron, J.-C.; Duvaldestin, P. Pharmacokinetics of alfentanil in children undergoing surgery. *British journal of anaesthesia* **1987**, *59*, 1437-1440.
96. Pokela, M.-L.; Ryhanen, P.T.; Koivisto, M.E.; Olkkola, K.T.; Saukkonen, A.-L. Alfentanil-induced rigidity in newborn infants. *Anesthesia & Analgesia* **1992**, *75*, 252-257.
97. Goresky, G.V.; Koren, G.; Sabourin, M.; Sale, J.P.; Strunin, L. The pharmacokinetics of alfentanil in children. *Anesthesiology* **1987**, *67*, 654-659.
98. Scierka, A.M.; Davis, P.J.; Killian, A.; Stiller, R.L.; Cook, D.R.; Guthrie, R.D. Pharmacokinetics of alfentanil in newborn premature infants and older children. *Developmental pharmacology and therapeutics* **1989**, *13*, 21-27.
99. Helmers, H.; Van Peer, A.; Woestenborghs, R.; Noorduin, H.; Heykants, J. Alfentanil kinetics in the elderly. *Clinical Pharmacology & Therapeutics* **1984**, *36*, 239-243.
100. Camu, F.; Gepts, E.; Rucquoi, M.; Heykants, J. Pharmacokinetics of alfentanil in man. *Anesthesia & Analgesia* **1982**, *61*, 657-661.
101. Phimmasone, S.; Kharasch, E.D. A pilot evaluation of alfentanil-induced miosis as a noninvasive probe for hepatic cytochrome P450 3A4 (CYP3A4) activity in humans. *Clinical Pharmacology & Therapeutics* **2001**, *70*, 505-517.
102. Fragen, R.; Booij, L.; Braak, G.; Vree, T.; Heykants, J.; Crul, J. Pharmacokinetics of the infusion of alfentanil in man. *British journal of anaesthesia* **1983**, *55*, 1077-1081.
103. Bovill, J.G.; Sebel, P.S.; Blackburn, C.L.; Heykants, J. The pharmacokinetics of alfentanil (R39209): a new opioid analgesic. *Anesthesiology* **1982**, *57*, 439-443.
104. Guay, J.; Gaudreault, P.; Tang, A.; Goulet, B.; Varin, F. Pharmacokinetics of sufentanil in normal children. *Canadian journal of anaesthesia* **1992**, *39*, 14-20.
105. Bovill, J.G.; Sebel, P.S.; Blackburn, C.L.; Oei-Lim, V.; Heykants, J.J. The pharmacokinetics of sufentanil in surgical patients. *Anesthesiology* **1984**, *61*, 502-506.
106. Taverne, R.H.; Ionescu, T.I.; Nuyten, S.T. Comparative absorption and distribution pharmacokinetics of intravenous and epidural sufentanil for major abdominal surgery. *Clinical pharmacokinetics* **1992**, *23*, 231-237.
107. Helmers, J.; Noorduin, H.; Van Peer, A.; Van Leeuwen, L.; Zuurmond, W. Comparison of intravenous and intranasal sufentanil absorption and sedation. *Canadian journal of anaesthesia* **1989**, *36*, 494-497.

108. Matteo, R.S.; Schwartz, A.E.; Ornstein, E.; Young, W.L.; Chang, W. Pharmacokinetics of sufentanil in the elderly surgical patient. *Canadian journal of anaesthesia* **1990**, *37*, 852-856.
109. Yan, Z.; Wu, X.-m.; Duan, J.-l.; Sheng, X.-y.; Wei, L.; Wei, L.; Zhang, L.-p.; Xu, C.-y. Pharmacokinetics of sufentanil administered by target-controlled infusion in Chinese surgical patients. *Chinese medical journal* **2009**, *122*, 291-295.
110. Borenstein, M.; Shupak, R.; Barnette, R.; Cooney, G.; Johnson, W.; Tzeng, T.-B. Cardiovascular effects of different infusion rates of sufentanil in patients undergoing coronary surgery. *European journal of clinical pharmacology* **1997**, *51*, 359-366.
